# A Complete Process of Text Classification System Using State-of-the-Art NLP Models

**DOI:** 10.1155/2022/1883698

**Published:** 2022-06-09

**Authors:** Varun Dogra, Sahil Verma, Kavita, Pushpita Chatterjee, Jana Shafi, Jaeyoung Choi, Muhammad Fazal Ijaz

**Affiliations:** ^1^School of Computer Science and Engineering, Lovely Professional University, Phagwara, Punjab, India; ^2^Department of Computer Science and Engineering, Chandigarh University, Mohali 140413, India; ^3^Bio and Health Informatics Research Lab, Chandigarh University, Mohali 140413, India; ^4^Machine Learning and Data Science Research Lab, Chandigarh University, Mohali 140413, India; ^5^Tennessee State University, Nashville, TN, USA; ^6^Department of Computer Science, College of Arts and Science, Prince Sattam Bin Abdul Aziz University, Wadi Ad-Dwasir 11991, Saudi Arabia; ^7^School of Computing, Gachon University, Seongnam-si 13120, Republic of Korea; ^8^Department of Intelligent Mechatronics Engineering, Sejong University, Seoul 05006, Republic of Korea

## Abstract

With the rapid advancement of information technology, online information has been exponentially growing day by day, especially in the form of text documents such as news events, company reports, reviews on products, stocks-related reports, medical reports, tweets, and so on. Due to this, online monitoring and text mining has become a prominent task. During the past decade, significant efforts have been made on mining text documents using machine and deep learning models such as supervised, semisupervised, and unsupervised. Our area of the discussion covers state-of-the-art learning models for text mining or solving various challenging NLP (natural language processing) problems using the classification of texts. This paper summarizes several machine learning and deep learning algorithms used in text classification with their advantages and shortcomings. This paper would also help the readers understand various subtasks, along with old and recent literature, required during the process of text classification. We believe that readers would be able to find scope for further improvements in the area of text classification or to propose new techniques of text classification applicable in any domain of their interest.

## 1. Introduction

In recent years, we have seen a growth in the amount of digital textual data available, which has generated new perspectives and so created new areas of research. With the emergence of information technology, the monitoring of such digital textual data is of great importance in many areas such as the stock market: gathering data from news sources to forecast the movement of underlying asset volatility [1], forecasting the stock prices of green firms in emerging markets [2], understanding the impact of tone of communications on stock prices [3], and determining indicators for stock prices volatility [4]; healthcare: disease surveillance [5, 6]; politics: developing a probabilistic framework on politics using short text classification [7]; education: understanding pedagogical aspects of the learners [8]; tourism: analyzing travelers sentiments [9]; and e-commerce: predicting success by evaluating users' reviews [10].

**Figure 1:**
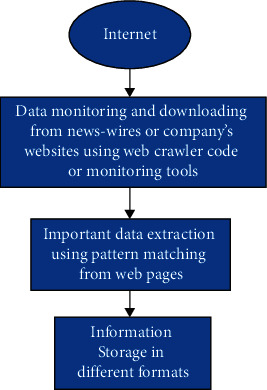
Monitoring and downloading relevant text documents to subject groups.

News is widely available in electronic format on the World Wide Web these days, and it has proven to be a valuable data source [11]. The volume of news, on the other hand, is enormous, and it is unclear how to use it most efficiently for domain-specific research. Therefore, a framework or architecture is required for a domain-specific news monitoring system, as well as a classification mechanism for classifying relevant online news into distinct subject groups automatically [5]. News monitoring is a type of oversight system that monitors and ensures the quality of each news instance generated, used, and retained for a purpose. Processes for assessing news to guarantee its completeness, consistency, and correctness, as well as security and validity, are included in these methods. There is always a need for a methodology that can extract meaningful information from a pool of textual documents belonging to distinct subject groups intended for certain research as shown in Figure 1.

Indeed, the majority of the digital data are available in the form of text, but this is usually unstructured or semistructured [12]. Thus, to make data useful for decision-making, structuring this textual data became a necessity [13, 14]. However, because of the high volume of data, it is quite impossible to process the data manually. Text classification has evolved due to this challenge. It is defined as assigning the text documents to one or more categories (called labels) according to their content and semantics. Traditionally, the majority of classification tasks were used solved manually, but it was expensive to scale. Classification can be thought of as writing rules for assigning a class to similar text documents. These rules include some related information that identifies a class. Handwritten rules can be performed well, but creating and maintaining them over time requires much manpower. A technical expert can frame rules by writing regular expressions that could maximize the accuracy of the classifier. The existing studies have proposed various techniques to automatically classify text documents using machine learning [15, 16]. In this approach, the set of rules or criteria for selecting a classifier is learned automatically from the training data. Under each class, it requires a lot of training documents and expertise to label the documents. The labeling is a process of assigning each document to its associated class. The labeling process was easier than writing handcrafted rules. Moreover, there exist variously supervised and semisupervised learning techniques that can even reduce the burden of manual labeling [17, 18]. This can be performed using automatic labeling. Automated text classification methods can be divided into three groups: rule-based methods, data-driven methods, and hybrid methods.

**Figure 2:**
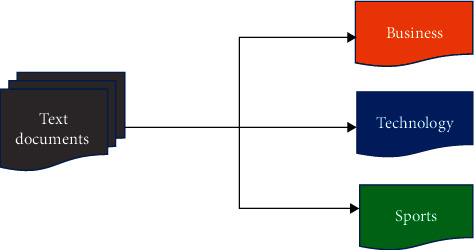
Labeling text documents with appropriate predefined classes or labels during the process of text classification.

Using a set of predefined rules, rule-based techniques classify text into various categories as shown in Figure 2. For example, the “fruit” label is applied to any document with the words “apple,” “grapes,” or “orange.” These techniques require a thorough knowledge of the domain, and it is difficult to maintain the systems. Data-driven methods, on the other hand, learn to make classifications based on previous data values. A machine learning algorithm can learn the inherent associations between pieces of text and their labels using prelabeled examples as training data. It can detect hidden patterns in the data, is more flexible, and can be applied to different tasks. As the title indicates, hybrid approaches use a mixture of rule-based and machine learning methods (data-driven) for making predictions.

In recent decades, models of machine learning have attracted a lot of interest [19, 20]. Most conventional models based on machine learning follow the common two-step method, where certain features are extracted from the text documents in the first step, and those features are fed to a classifier in the second step to make a prediction. The popular feature representation models are BOW (bag-of-words), TF-IDF (term frequency-inverse document frequency), and so on. And the common classifiers are naïve Bayes, KNN, SVM, decision trees, random forests, and so on. These models are discussed in detail in the following sections. Deep learning models have been applied to a wide variety of tasks in NLP, improving language modeling for more extended context [21–23]. These models are attempting, in an end-to-end fashion, to learn the feature representations and perform classification. They not only have the potential to uncover latent trends in data but also are far more transferable from one project to another. Quite significantly, in recent years, these models have become the mainstream paradigm for the various tasks of text classification. The following are some of the natural language challenges solved with the text classification.

*Topic modeling* is widely used to extract semantic information from text data. An unsupervised method of topic modeling learns the collection of underlying themes for a batch of documents as well as the affinities of each document to these topics.

*News classification*: online news reporting is one of the most significant sources of information. The task of finding and deriving structured information about news events in any text and assigning the relevant label is referred to as news classification.

*Sentiment classification* is an automatic technique of discovering views in text and classifying them as negative, positive, or neutral based on the emotions expressed in text. Sentiment classification, which uses NLP to evaluate subjective data, can help understand how people think about company's products or services.

*Question answering* has rapidly evolved as an NLP challenge that promises to deliver more intuitive means of knowledge acquisition. In contrast to the typical information retrieval approach of creating queries and perusing results, a question answering system simply takes user information requests stated in ordinary language and returns with a brief answer.

*Language translation* models have been attempting to translate a statement from one language to another resulting in perplexing and offensively inaccurate results. NLP algorithms through text classification may be trained on texts in a variety of languages, allowing them to create the equivalent meaning in another language. This approach is even applicable to languages such as Russian and Chinese, which have historically been more difficult to translate due to differences in alphabet structure and the use of characters rather than letters, respectively.

Nevertheless, it is observed that most text classification literature studies for solving NLP challenges are limited to showcasing the results of text classification using standard or state-of-the-art methods and focusing on specific research domains. For example, the authors mention the application of text analytics in the industry, but the task of monitoring and collecting text data was not detailed, and the scope of the proposed models appeared limited to particular domains [24]. In another study, the authors discuss the information extraction from tweets for monitoring trucks fleets to model truck trips, but it does not cover the feature selection or extraction methods to achieve information extraction [25]. Other studies [26, 27] focus on text classification for domain-specific search engine based on rule-based annotated data; however, it does not cover the semisupervised or unsupervised approaches of labeling data to achieve text classification [28]. Moreover, these works do not reveal the latest techniques being used in the area of natural language processing. The deep learning-based pretrained language representation model can be explored in information extraction and classification. These studies also lack in detailing the subtasks require to initiate the research in text classification, that is, data collection, data preprocessing, and semisupervised or unsupervised data labeling for training machine learning models. To the best of our knowledge, there are no similar review studies available that cover in-depth presentations of various subtasks of text classification.

**Figure 3:**
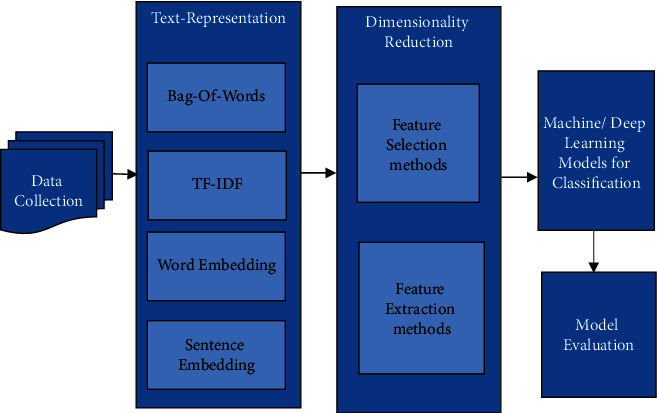
Subtasks of the text classification process cover state-of-the-art data collection, text representation, dimensionality reduction, and machine learning models for classifying text documents to an associated predefined class/label.

In this paper, we focus to overcome the above-mentioned issues. We put a lot of effort to create qualitative research for text classification to help us understand its subtasks or elements. Moreover, this paper presents the old and latest techniques used in each subtask of text classification as shown in Figure 3 along with their benefits and limitations. It also presents the research gap in the area of text classification by examining various existing studies. The key contribution of the study is mentioned below:

- Discussing the subtasks of text classification
- Presenting the most recent and former techniques used in each subtask
- Presenting benefits and limitations of various models used in the process of text classification
- Presenting the research scope for further improvements in existing techniques and proposing new techniques with their application in different domains

Section 2 presents the process of text classification along with the comprehensive literature on each subtask; Section 3 presents the evaluation methods of classification techniques; Section 4 presents the comparison of approaches or models used in the subtasks of the text classification system mentioning their benefits and limitations; Section 5 presents the research gap and further scope for research; and Section 6 concludes the existing studies.

## 2. Text Classification: Framework

Text classification is a problem formulated as a learning process where a classifier is used to train to differentiate between predefined classes based on features extracted from the collection of text documents [29]. The accuracy of the classifier depends upon the classification granularity and how well separated are the training documents among classes [30, 31]. In text classification, a set of labels or classes are given, and we need to evaluate which class/label a particular text document relates to. Usually, a class or label is a general topic such as sports or business. But it may be significantly more difficult to distinguish between documents that are about more similar classes such as networks and the internet of things. Certain features represent the potential overlap between classes; the learning task would be simplified by removing such overlapping features. If the gap between classes could be increased, the classification performance would increase. This can be achieved through features weighting and selecting valuable features. Text classification has been studied and applied by many researchers in real-world scenarios such as sentiment classification of stock market news and its impact [31], news classification for syndromic surveillance [5], microblog topic classification [32], domain adaptation for sentiment classification [33, 34], and brand promotion based on social media sentiments [35, 36].

**Figure 4:**
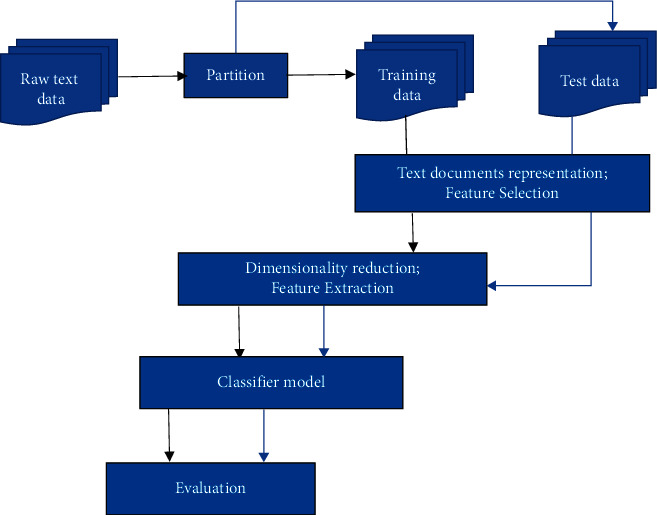
A text classification framework. Note: Black connecting lines represent training and blue connecting lines represent the testing phase.

The text classification process is described as classifying a set of *N* documents; first, we build a classifier *T*. There is a collection of text documents *D*, and every text document is given a class/label by an expert. Secondly, we need to train a classifier for each class/label by giving input as a corresponding set of documents in *D*. Now we need to apply trained classifier *C* to classify *N* documents. We will get each document in *N* assigned to a predefined class/label by *C*. Text classification is a comprehensive process that includes not just model training but also several other steps including data preprocessing, transformation, and dimensionality reduction. The process starts with the collection of textual content from various sources. The textual content may be belonging to a domain(s) representing some events, business processes, or public information. Then these text documents require preprocessing to generate appropriate text representation for the learning model. This is done in two phases: in phase 1, the features are extracted from the processed text using any feature extraction algorithm, and in phase 2, the features are reduced by applying feature selection techniques. This reduction of features tends to decrease the dimensions of data required for the learning method. After these phases, the learning algorithms are chosen to train on data to generate the best classifier for recognizing a target category or class. This text data required to train a classifier is known as training data. The data is divided into two sets: the majority of data are taken for the training model, and the rest part of the data is taken for testing the classifier, known as testing data. Similarly, the model is trained to recognize each target class representing its data available in the associated text documents. During the testing phase, when a classification method is developed, it is executed on test data to define the target class of input text, and the result is produced in the form of weights or probabilities. Finally, the result is evaluated for its accuracy, of text classifier, using evaluation techniques. These are the main phases or subtasks of the text classification process, also shown in Figure 4. The different approaches have been used in each phase of text classification discussed in the next subsections of the study.

### 2.1. Data Collection

The initial stage in text classification is to acquire text data from different sources as per the research domain. There are several online open data sets available, for example, various newsgroups (Bloomberg, Reuters, Financial Express), Kaggle, and WebKB for solving a classification problem. Researchers have used such database architecture for their research purposes [37–39]. The corpus can also be built with data that could be anything from emails, language articles, company's financial reports, medical reports, to news events. In the study, the authors have created a fine-grained sentiment analysis corpus for annotating product reviews. However, they faced the most challenging tasks that had not been targeted in applications such as sentiment analysis, target-aspect pair extraction, and implicit polarity recognition, for recognizing aspects and searching polarity with nonsentiment sentences [40].

### 2.2. Text Document Representation: Features Construction and Weighting

Text classification is the most demanding area of machine learning for understanding texts written in any natural language. One of the most essential tasks that must be completed before any classification process is text representation. Moreover, the texts cannot be provided as input to the machine learning models because almost all algorithms take input in numbers as feature vectors with a predefined size instead of the textual data with variable length. To resolve this issue, first textual data need to be transformed to document vectors. This can be done in two different ways in general. The first is a context-independent approach in which a document is represented as a set of terms with their corresponding frequency in the document, but they are independent of the sequence of terms in the collection. The second approach is to represent text as strings, with each document consisting of a sequence of terms. The following subtopic covers the various representations in natural language processing from the early days to the latest state-of-the-art models.

#### 2.2.1. Context-Independent Approaches

**Figure 5:**
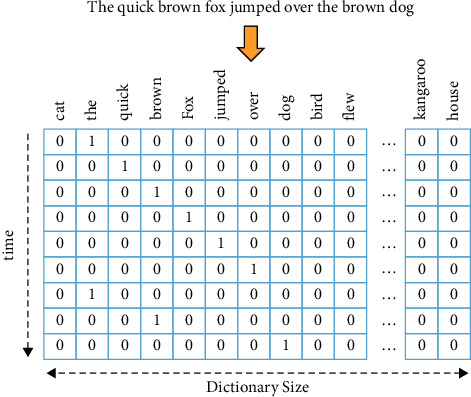
One-hot representation, a tensor that is used to represent each document. Each document tensor is made up of a potentially lengthy sequence of 0/1 vectors, resulting in a massive and sparse representation of the document corpus [43].

The bag-of-words [41] is the most commonly used model in document transformation that considers every word in the text document as a token/feature although words' order and their context are ignored. Each word, sometimes tens or hundreds of dimensions, is represented by a real-valued vector called a one-hot representation [42]. The feature vector has the same length as the vocabulary size, and only one dimension is on as shown in Figure 5. However, the one-hot representation of a word suffers from data sparsity. On the other hand, the words with very high frequency may cause biases and dominate results in the model [44]. To overcome the weaknesses of BOW, the text documents are represented with weighted frequencies, a document-term matrix, where a column signifies a token and a row signifies a document. This scheme of assigning weights to token frequencies in the form of a matrix is called TF-IDF (term frequency-inverse document frequency). During the implementation of this model using a matrix, the value *w*_*ij*_ in each cell corresponds to the weight of *t*_*j*_ in *d*_*i*_ that is calculated as *tf* ⟨*t*_*j*_, *d*_*i*_ │ *n*_*d*_*i*__⟩, where *tf* ⟨*t*_*j*_, *d*_*i*_⟩ represents the count of token *t*_*j*_ in text document and *d*_*i*_ and *n*_*d*_*i*__ represents the total quantity of token *t*_*j*_ in document *d*_*i*_. Due to the simplicity of the model, this is preferably used in natural language processing. The improved features subset using this approach has been taken together with the characteristics of term frequency and document frequency [45–47]. However, even a small collection of documents may consist of a large number of meaningful words that leads to the problem of scalability or high dimensionality. This offers opportunities to find effective ways to decrease running time or reduce high dimensionality in the case of a large number of documents.

The primary alternative has emerged in the form of statistical language modeling for modeling complex text classification or other natural language tasks. In its beginning, however, it used to struggle with the curse of dimensionality when studying typical probability functions of language models [48]. This led to the inspiration to learn distributed representations of low-dimensional space terms. The distributed representations describe a co-occurrence matrix of terms × terms that considers the frequency of each term that appears in the context of another term, with a window size of *k* [49]. The singular value decomposition was used for text representation, where the matrix decomposition technique was used for reducing a given matrix to its constituent matrices via an extension of the polar decomposition with the idea of making subsequent matrix calculations simpler. It gives the top rank-k constituent parts of the original data. The singular value decomposition will break this into best rank approximation capturing information from most relevant to least relevant ones [49].

**Figure 6:**
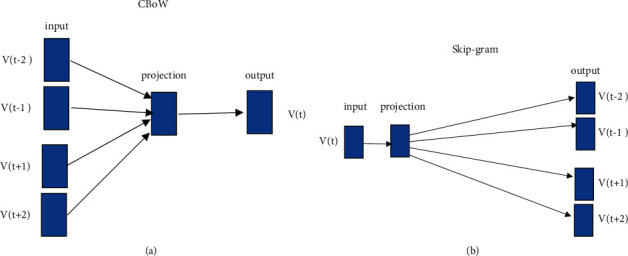
The word2vec algorithm uses two alternative methods: (a) continuous bag of words (CBoW) and (b) skip-gram (SG) [50].

The big popularization of word embedding was possibly due to the continuous bag-of-words (CBOW) paradigm to create high-quality distributed vector representations effectively. A solution is designed to counter the curse of dimensionality where a distributed representation for each word is concurrently studied along with the probability distribution for word sequences represented in terms of such representations [21]. The continuous bag-of-words is a prediction-based model that directly learns word representation as shown in Figure 6(a). The distributed representations of context (or surrounding words) are combined in the CBOW model to predict the word in the middle. The CBOW has reshaped the word embedding [51]. The continuous bag of representation is applied with a neural network model to achieve improved accuracy in classification [52, 53]. Another model is designed called skip-gram that further reshaped the word embedding [54]; its architecture works in reverse of what the continuous bag-of-words model does. The model predicts each context word from the target word as shown in Figure 6(b). It iterates on the words of each sentence in the given corpus and uses the current word to predict its neighbors (its context); thus, the model is called “skip-gram” (local context window) [55].

Weighted words calculate document similarity directly from the word-count space, which takes longer to compute for large vocabularies. While counts of unique words give independent evidence of similarity, semantic similarities between words are not taken into consideration. Word embedding techniques solve this problem, but they are constrained by the need for a large corpus of text data sets for training. The word embedding algorithms were developed using the word and its closest neighbor. There was an approach suggested by authors for generating a word embedding GloVe (global vector, combines count- and predict-based methods) model for distributed word representation. The unsupervised learning algorithm, where a model is trained on overall statistics of word-word co-occurrence that how often it appears in a corpus, and the result obtains the vector representation of words with linear substructures of the word vector space [56]. GloVe's solution is to count how many times a term *i* (context word) in another term *j* (target word) occurs. The purpose is to establish a meaning for the word *i* and word *j* as to whether the two words occur close to *N*-word apart or not. The encoding vector includes the ratio of two words specifically recognized as a count-based system of co-occurrence probabilities. The prediction-based approach receives popularity, but GloVe's authors claim that the count-based methodology incorporates the global statistics and may be more effective because it outperforms word representation testing on word comparison, term similarity, and called entity recognition tasks.

The enhanced text document representation system was developed to work on the issues of traditional feature-based extraction techniques that included only nouns and nouns phrases to represent the important events called event detection techniques [57]. This technique has used fewer tokens or features than bag-of-words to handle the problem of scalability or high dimensionality of documents. Furthermore, this technique has led to another representation based on named entities. The authors have presented the classification of tweets using named entity recognition to filter out noiseless required information [30, 58]. It works by finding proper nouns in the documents that belong to predefined categories. This process involves systematically assigning categories to each term or entity while developing a corpus or labeling process. But this corpus-based representation was unable to represent some domain-specific words that are infrequent during training. The authors have proposed techniques to deal with infrequent or unseen words during labeling [59].

Previous representations were not considering the morphological relation of words to disambiguate the unseen words. Many studies have presented methods that automatically extract features from the documents. These have used infrequent words that produce a high variety of low anticipated relations between the text documents. This kind of information once aggregated provides potentially less obvious and hidden relations in the text documents. Using less-frequent words with lexical constraints has reduced the associated cost of knowledge re-engineering, and it was able to process many documents from a large number of domains [59, 60]. These methods help for better representations of text documents especially handling unseen or less-frequent words. And the problem of scalability was also controlled and associated with word's semantical approach. There is another approach that was proven most efficient in a domain-specific text representation, *proper nouns*, an intermediate solution between noun phrases and named entities. This technique has reduced the ambiguity that occurred due to particular associated nouns with more than one named entity category [31]. Recent approaches are concentrating on capturing context beyond the word level to produce performance by giving a more structured and semantic notion of text [61].

#### 2.2.2. Context-Aware Approaches

Context-aware classification approaches essentially find and employ term association information to increase classification effectiveness. They allow the presence or absence of a term to impact how it contributes to a classification outcome. Context is a concise term referring to high-level semantics. It may be taken in several ways and used in a variety of dimensions. We categorize context-based classification systems according to how the context was understood and what features were used to determine it.

The authors have come up with improved embedding for texts, such as *word2vec*, which transforms a word into an *n*-dimensional vector. To map the words into an Euclidean space, we can go through an approach to creating sequence embedding that brings a sequence into an Euclidean space. One of the sequential data function learning problems is called sequence embedding [62], where the aim is to convert a sequence into a fixed-length embedding. This approach is a highly strong tool for identifying correlations in text corpora as well as word similarity. However, it falls limited when it comes to capturing out-of-vocabulary words from a corpus. *RNN-*based models interpret the text as a sequence of words and are intended for text classification to capture word dependencies and text structures [63]. By adding a memory cell to remember values over arbitrary time intervals and three gates (input gate, output gate, and forget gate) to control the flow of information into and out of the cell, *LSTM* addresses gradient vanishing or exploding problems experienced by the RNNs. In a recursive method, the authors expand the chain-structured LSTM to tree structures, using a memory cell to store the background of multiple child cells or multiple descendants. They claim that the new model offers a realistic way to understand contact between hierarchies between long distances, for example, language parse structures [64]. To capture text features, the authors also incorporate a *bidirectional-LSTM* (Bi-LSTM) model with two-dimensional max-pooling [65]. The seq2seq model is used in various NLP applications [66, 67]. Most real-world problems have a data set with a substantial number of unusual words. The embeddings learned from these data sets are unable to produce the correct representation of the word. To do this, the data set needs to have a large vocabulary. Words that appear frequently help you create a large vocabulary. Second, when learning embeddings from scratch, the number of trainable parameters grows. As a result, the training process is slowed. Learning embeddings from scratch may also leave you confused about how the words are represented [68].

**Figure 7:**
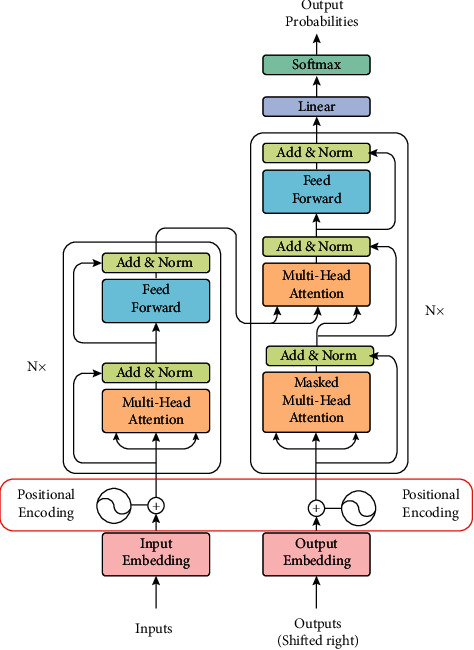
The transformer architecture.

Pretrained word embeddings are the solution to all of the above difficulties. The studies have consistently shown the importance of transfer learning by pretraining a neural network model on an established problem in the field of computer vision and then doing fine-tuning utilizing the learned neural network as the foundation for a new purpose-specific model. It is demonstrated in recent years that a related approach may be effective in several tasks relating to natural language. This is another kind of word embedding; the classification algorithms provide a greater sense of learning the features of such embeddings. Yet such embeddings do not take the word order or the word meaning of each sentence into consideration. This is where *ELMo* (embedding from models of language) comes into action. ELMo is a contextual embedding that takes into consideration the terms that surround it. It models word use characteristics such as morphology and how it is used in different contexts. The term vectors are learned features of a deep bidirectional language model (biLM) internal state that is pretrained on a broad text corpus. The authors have demonstrated that these representations can be readily applied to current frameworks and greatly strengthen the state-of-the-art NLP issues such as addressing queries, textual entailment, and interpretation of emotions [62]. A Transformer [69] is another solution to working with long dependencies such as LSTM. LSTM is long short-term memory, a sort of neural network that has a “memory unit” capable of maintaining knowledge in memory over strong periods helping it to learn longer-term dependencies [22]. The Transformer is based through the encoder and decoder on an attention process as shown in Figure 7. The Transformer allows the use of this method to store knowledge about the specific meaning of a given term in its word vector.

Unlike past deep contextualized language representation studies [62] that take into account the backward model as in the ELMo bidirectional LSTM, the authors proposed a new type of language representation named *BERT*, which stands for bidirectional transformer encoder representations. BERT uses a Transformer, a mechanism of *attention* that learns contextual connections between words (or subwords) in a document. The authors have argued that conventional technologies limit the power of the pretrained representations, especially for the approaches to fine-tuning [70]. The main constraint is the unidirectional existence of modern language models, which restricts the range of frameworks that can be used during pretraining. BERT is structured to pretrain profound bidirectional representations from unlabeled text documents by jointly conditioning across both layers in both the left and right contexts [71]. In setting language modeling, transformers can acquire longer-term dependence but are constrained by a fixed-length context. The authors suggested a novel Transformer-XL neural architecture that allows learning dependence to interrupt temporal coherence beyond a fixed length. It consists of a recurrence function at the segment level and a novel positional encoding scheme [69]. Learning regarding the inductive transfer has significantly impacted computer vision, but current techniques in NLP also need complex task modifications and preparation from scratch. The authors suggested universal language model fine-tuning (ULMFIT), an important transfer learning approach that can be extended to any NLP task, and implemented techniques that are essential to fine-tuning a model of language [72].

A total of 15% of the words in every sequence are substituted with a mask token before feeding word sequences into BERT. The model then tries to determine the actual context of the masked words in the list, based on the context given by the other, unmasked, words. The BERT loss function only considers the estimation of the masked terms and excludes the estimation of the unmasked phrases. As a result, the model converges slower than directional ones, a feature offset by its enhanced understanding of the context. Centered on BERT's masking technique [71], the authors developed a novel language representation model enhanced by knowledge-masking technique named *ERNIE* (enhanced representation by knowledge integration) [73], which involves masking at the entity level and masking at the phrase level. Strategy at the entity level covers entities that typically consist of several terms. The phrase-level technique covers the whole phrase consisting of many words that serve as a cohesive entity together. Their experimental findings indicate that ERNIE outperforms other standard approaches by obtaining modern state-of-the-art outcomes on natural language processing activities, including natural language inference, conceptual similarity, named-entity identification, emotion analysis, and question answering. DistilBERT, a technique for pretraining a smaller general-purpose language representation model that can later be fine-tuned with high performance on a wide range of tasks like its bigger equivalents, is created. While most previous research focused on using distillation to build task-specific models [74, 75], this study uses knowledge distillation during the pretraining phase and demonstrates that it is possible to reduce the size of a BERT model by 40% while retaining 97% of its language understanding capabilities and being 60% faster [76].

The Transformer paradigm is generally popular in several tasks relating to natural language processing. Using a transformer is therefore also an expensive operation since it requires the method of self-attention. The Transformer employs an encoder-decoder design that includes stacked encoder and decoder layers. Two sublayers comprise encoder layers: self-attention and a positionwise feed-forward layer. Self-attention, encoder-decoder attention, and a positionwise feed-forward layer are the three sublayers that comprise decoder layers. Self-attention assumes we are conducting the task of attention on the sentence itself, as compared to two separate sentences. Self-attention allows defining the connection in a single sentence between the words. It is the function of self-attention that adds to the expense of utilizing a transformer. The quadratic structure of self-attention, however, constrains its operation on long text. Attention is described using the (query, key, and value) model. A query *Q* is a “context,” and in previous equations, the prior concealed state is employed as the query context. Based on what we already know, we want to know what happens next. The value represents the features of the input. The phrase “key” is just an encoding of the word “value.” To attract attention, the query's relevancy to the keys is established. The associated values that are unrelated to the query are then hidden. The authors follow a method of fine to coarse attention on multi-scale spans by binary partitioning (BP); they suggest *BP*-*Transformer*. BP-Transformer has a strong balance between the complexity of computations and the capability of models. The authors performed a series of experiments on text classification and language processing, showing that BP-Transformer performs superior to previous self-attention models for long text [77]. The Binary-Partitioning Transformer attempts to boost the self-attention mechanism's usefulness by considering the transformer as a graph neural network. Any node in this graph represents an input token.

Another research suggests a variant of the Neural Attentive Bag-of-Entities, which is a neural network algorithm that uses entities in a knowledge base to conduct text classification. Entities include unambiguous and specific syntactic and semantic signs that are useful for catching semantics in documents. The authors put together easy high-recall dictionary-based entity recognition, with a neural attention system that helps the model concentrate on a limited number of unambiguous and specific entities in a text [78]. The model first identifies entities to whom this name might be addressed (e.g., Ap Inc., Apple (food)) and then describes the entity using the weighted average of all entities' embedding. The weights are measured using a modern method of neural attention that helps the model concentrate on a specific subset of entities that are less ambiguous in context and more important to the document.

It is the time for NLP when the transition started as we listed the unsupervised pretrained language models that had made breakthroughs in different tasks of understanding the natural language, such as named-entity identification, emotion interpretation, and question-answer records for art performance beginning one after another in that short period. These NLP models indicate that a lot more is yet to come, and the authors look forward to researching and implementing them. However, the authors proved that a single pretrained language model may be applied as “a zero-shot task transfer” to execute basic NLP tasks without the requirement for fine-tuning on a training example data set. While this was an encouraging proof of concept, the best case performance only equaled certain supervised baselines on a single data set. Performance on most tasks was still well behind even simple supervised baselines. Across one order of magnitude of scaling, the study found generally consistent log-linear trends in performance on both transfer tasks and language modeling loss. GPT-3 performs well on NLP tasks in the zero- and one-shot settings and, in the few-shot setting, is sometimes comparable with, and occasionally surpasses, the state of the art (although the state-of-the-art is held by fine-tuned models). This might imply that bigger models are better meta-learners [79].

GPT-3 approaches the performance of a fine-tuned RoBERT as a baseline on the “Challenge” version of the data set, which has been filtered to questions (multiple-choice questions on common sense reasoning collected from 3rd to 9th-grade science examinations) that standard statistical or information retrieval algorithms are unable to accurately answer. GPT-3 marginally outperforms the same fine-tuned RoBERT baseline on the “Easy” version of the data set. However, both of these findings are much inferior to MBART's overall SOTAs.

### 2.3. Data Preprocessing: Data Cleaning

There are a lot of text data available today, and data are being grown daily in structured, semiunstructured, or fully unstructured forms. To perform the text classification task, it is always required to process the raw corpus data. There are many steps involved in data processing; generally, data cleaning, that is, organizing the data as per the structure and removal of unneeded subtexts; tokenization, that is, breaking up text into words; normalization, that is, converting all texts into the same case, removing punctuation (stemming leaves out root forms of the verb and lemmatization); and substitution, that is, identifying candidate words for translation, performing word sense disambiguation [80]. In one of the studies [81], the researchers have also focused on how machine learning techniques are needed to design to recognize similar texts when text data are downloaded from multiple and heterogeneous resources. In the labeling task, the text documents are labeled with two commonly used approaches; one is to label each part of the text individually, and the second is to label the group of texts. The first approach includes different supervised learning methods, and the second is called multi-instance learning [82, 83].

### 2.4. Data Preprocessing: Dimensionality Reduction

Dimensionality reduction is a crucial approach in data preprocessing for preparing data for text classification. It is done to reduce the classifier's memory requirements and execution time, hence increasing the learning model's efficiency and efficacy. The dimensions of data are increasing as the volume of data grows. To map large dimensions to space with low dimensions, it becomes necessary to reduce the dimensions of the data [84, 85]. The purpose of decreasing high-dimensional space is to find a subdimensional space that is less complex and can adjust the learning model to the greatest extent possible. In some cases, several researchers have noticed that the number of features in samples is substantially larger than the number of samples. This leads to a problem known as overfitting [86]. As a result, dimensionality reduction becomes necessary to avoid overfitting. Feature selection and feature extraction are two significant subtasks in lowering dimensionality. The process of supplying the part of the original attributes that are necessary for the task is known as feature selection. Feature extraction is a technique for changing space with many dimensions into a new space with few dimensions, to increase data variance [87].

#### 2.4.1. Standard Feature Selection Methods

The following are the goals of the feature selection method:

- To improve the predictability of the classifiers
- To create a cost-effective classifier while also speeding up the process
- To improve the clarity of the data-gathering approach

The approach of finding a portion of the original attributes that are significant for the training set is known as feature selection. It is used to create an effective classifier while keeping important lexical properties in vocabulary [88]. It removes the noisy attributes that tend to reduce accuracy [89]. The goal of feature selection is to decrease the feature space by picking a subset of Kay attributes and minimizing overfitting while maintaining text classification performance [90]. For instance, a feature or attribute set *X*={*X*_1_,  *X*_2_,…., *X*_*N*_}

When *N* is a group of feature sets, 2*N* feature subsets are created, each of which is represented by a vector of size *N*. The methods identify a feature subset of size *K*, *K* < *N* without losing the accuracy of the full feature set. It has been the subject of investigation, and the writers have so far provided many techniques. Filter, wrapper, and embedded are the three types of approaches available [91, 92]. Filter-based feature selection offers several ways to evaluate the information value of each feature. The filter approach picks the top-*N* features based on the results of various statistical tests to identify a connection with the target label or to establish which attributes are more predictive of the target label, and it is independent of any learning algorithms. Because this technique ignores feature dependencies, it is computationally efficient. The wrapper technique evaluates a subset of features based on their utility to a given class. It uses a learning model to assess the subset of features based on their predictive power, but it is significantly very costly owing to repetitive learning and cross-validation. Embedded techniques are analogous to wrapper methods, except that they include feature selection during the training phase [93].

*(1)* Filter methods

*Univariate Feature Selection*. To determine the link between the features and the target variable, univariate feature selection evaluates each feature independently. Because they pertain to linear classifiers created using single variables, the following univariate feature selection methods are linear.
The filter approach chooses attributes without focusing on the core goal of improving any classifier's performance. To score the attributes, it uses the data's most important attributes. If *d* features or attributes are identified as *S*, the goal of a filter-based approach is to pick a subset of *m* *<* *d* features, *T*, which maximizes some function *F*:
  (1) $$\begin{array}{c} \tau^{*}={\text{argmax}}_{\tau ⊑S}F\left(\tau \right),\text{s.t.}\left|\tau \right|=m. \end{array}$$
It finally settles on the top-*m* rated features with the highest scores. This number is known as joint mutual information, and maximizing it is an NP-hard optimization problem since the number of potential feature combinations rises exponentially.
The following are the often used linear univariate filter techniques in text classification:
The *information gain* approach selects features based on the item's frequency concerning the class/label prediction. Researchers have demonstrated that by removing superfluous features without modifying the features, the approach may lower the vector dimensionality of text and enhance classification results [94]. It adds the most value when the text corresponds to a certain label or class, and the word is also present in the document. It can be written as follows:
  (2) $$\begin{array}{c} \text{IG}\left(t\right)=-\sum_{i=1}^{m}P\left(C_{i}\right)\log p\left(C_{i}\right)+p\left(t\right)\sum_{i=1}^{m}P\left(C_{i}|t\right)\log p\left(C_{i}|t\right)+p\left(\bar{t}\right)\sum_{i=1}^{m}P\left(C_{i}|\bar{t}\right)\log p\left(C_{i}|\bar{t}\right). \end{array}$$
The utility of feature *t* in the classification is measured by this formula. If *IG* is higher than the prior value without the feature *t*, the current feature *t* is more relevant for classification. In other words, the discriminating power of the term *t* increases as the value of the information gain *IG* increases. Here, *IG* stands for information gain; *C*_*i*_ is the *i*-th class; *P(C*_*i*_) is the probability of an *i*-th class; and *m* is several target classes. *P(t)* is the probability the feature *t* appears in the documents and the probability $P\left(\bar{t}\right)$ for feature; *t* does not appear in the document. *P(C*_*i*_*|t)* is the conditional probability of the feature *t* appearing in *i*-th class. $P\left(C_{i}|\bar{t}\right)$ is the conditional probability of the feature *t* that does not appear in *i*-th class.
The *Chi-square test* is a statistical strategy for assessing the relationship between a set of categorical features using their frequency distribution and determining how much the findings differ from the predicted output [95, 96]. This may be determined given events *A* and *B*, which are considered to be independent if
  (3) $$\begin{array}{c} p\left(AB\right)=p\left(A\right)p\left(B\right). \end{array}$$
The occurrence of the term and the occurrence of the class are the two events in feature selection. The terms are then ranked according to the following value. Chi-square can be calculated from the following equation:
  (4) $$\begin{array}{c} {\text{CHI}}^{2}\left(t,C\right)=\sum_{t\epsilon \left\{0,1\right\}}.\sum_{C\in \left\{0,1\right\}}\frac{{\left(N_{t,C}-E_{t,C}\right)}^{2}}{E_{tC}}, \end{array}$$
where *t* denotes the feature, *C* denotes the specific class, *N*_*t*,*C*_ is the frequency of feature *t* and class C occurring together, and *E*_*t*,*C*_ is the frequency of feature *t* occurring without class *C*. The chi-square between each feature and class is computed, and the features with the highest chi scores are chosen.
*Fisher score* calculates the variance of the predicted value from the actual value to get the information score or how much knowledge one variable has about the unknown parameter on which the variable depends, and when the variance is the smallest, the information score is the highest. For a support-vector-based feature ranking model, researchers employed Fisher's linear discriminant [97–99].
For instance, let *µ*_*j*_^*k*^ and *σ*_*j*_^*k*^ be the mean and standard deviation of the *k*-th class, concerning the *j*-th feature. Let *μ*^*j*^ and *σ*^*j*^ represents the mean and standard deviation of the entire training data concerning the *j*-th feature. The Fisher equation for determining the *j*-th feature's score is stated as follows:
  (5) $$\begin{array}{c} F\left(x^{j}\right)=\frac{\sum_{k=1}^{c}n_{k}{\left(\mu_{k}^{j}-\mu^{j}\right)}^{2}}{\sigma_{j}^{2}}, \end{array}$$
where *σ*_*j*_^2^ is computed as ∑_*k*=1_^*c*^*n*_*k*_(*σ*_*k*_^*j*^)^2^. Top-*m* features with higher fisher scores are chosen by the algorithms.
*Pearson's correlation coefficient* is used to measure linear dependency between two continuous variables by dividing their co-variance by the product of their standard deviation, and its value ranges from −1 to +1. In two variables, *a*–1 value signifies a negative correlation; *a* +1 value shows a positive correlation; and a 0 value represents no linear association [93].
Using vectors, Pearson's coefficient *r* can be computed as follows:
  (6) $$\begin{array}{c} r=\frac{\left(x_{1}-{\bar{x}}_{1}L\right)\left(x_{2}-{\bar{x}}_{2}L\right)}{\left(\left|x_{1}\right|\left|x_{2}\right|\right)}, \end{array}$$
where ${\bar{x}}_{1}$ is the mean of the vector *x*_1_ and similarly for *x*_2_, *L* is the vector of 1s, and |*x*| is the magnitude of vector *x*.
Variance threshold is a technique for reducing vector dimensionality by deleting all low-variance features. Features that have a lower training-set variance than the threshold will be deleted [100, 101].
  (7) $$\begin{array}{c} s=\frac{\sqrt{\sum {\left(x_{i}-\bar{x}\right)}^{2}}}{\left(n-1\right)}. \end{array}$$
The equation may be used to find features that have a variation below a given threshold. When the feature does not vary much within itself, it is seen to have low predictive potential.
*Multi-Variate Filter Methods*. During the assessment of the multi-variate filter selection approach, the interdependencies of features are also taken into account to choose relevant features.
It is based on mutual information that discovers the features in a feature set with the highest dependency with the target label. However, it is not appropriate for use when the goal is to achieve high accuracy with a small number of features.
Alternatively, it may utilize max relevance, which detects features with a dependency by averaging all mutual information values between all features *x*_*i*_ and target label *c*. *S* refers to features, and *I* represents mutual information; in the following equation, it is calculated between feature *i* and class *c*:
  (8) $$\begin{array}{c} \max D\left(S,c\right),D=\frac{1}{\left|S\right|}\sum_{x_{i}\in S}\left(I\left(x_{i;}c\right)\right). \end{array}$$
However, this results in a high level of redundancy, that is, a higher level of a dependency across features. As a result, to locate mutually exclusive features, minimum redundancy can be used [102].
  (9) $$\begin{array}{c} \min R\left(S\right),R=\frac{1}{{\left|S\right|}^{2}}\sum_{x_{i,}x_{j}\in S}I\left(x_{i},x_{j}\right), \end{array}$$
where *I*(*x*_*i*_,*x*_*j*_) is the mutual information between feature *i* and *j*.
*Multi-Variate Relative Discriminative Criterion*. The author offers a multi-variate selection strategy that takes into account both feature relevance and redundancy in the selection process. The RDC is used to assess the relevance, whereas Pearson's correlation is used to assess redundancy between features [103]. This measure boosts the rankings of terms that are exclusively found in one class or whose term counts in one class are much higher than in the other.
  (10) $$\begin{array}{c} \text{RDC}\left(w_{i},tc_{j}\left(w_{i}\right)\right)=\frac{\left(\left|\text{d}f_{\text{pos}}\left(w_{i}\right)-\text{d}f_{\text{neg}}\left(w_{i}\right)\right|\right)}{\text{min}\left(\text{d}f_{\text{pos}}\left(w_{i}\right),\text{d}f_{\text{neg}}\left(w_{i}\right)\right)*tc_{j}\left(w_{i}\right)}, \end{array}$$
where d*f*_pos_(*w*_*i*_), d*f*_neg_(*w*_*i*_) are the collection of positive and negative text documents, respectively, in which the term *w*_*i*_ is occurred. The word may be repeated several times in specific documents and represented by  *tc*_*j*_(*w*_*i*_). Instead of adding together RDC values for all term counts of a term, the area under the curve (AUC) for a difference graph is treated as term rank.
Researchers have been looking for novel approaches to increase classification accuracy while also reducing processing time. The author has provided a differentiate feature selector, a filter-based strategy that has picked unique features that have term properties while eliminating uninformative ones [92]. It provided efficiency by reducing processing time and improving classification accuracy.

*(2) Wrapper Methods.* Wrappers' approaches are bound to a certain classifier; the methods choose a subset of features based on their influence on the classifier by assessing the prediction performance of all potential feature subsets in a given space. It signifies that the features subset will be assessed by interacting with the classifier, which will improve the classification technique's accuracy. As the feature space expands, the computing efficiency suffers as a result of this method. Wrappers are used to choose features for other models as filters. The procedure may be accomplished in three ways: the first methodology employs a best-first search technique; the second methodology employs a stochastic approach such as random selection; and the third methodology use heuristics such as forward and backward passes to include and omit features.

 
*Multi-Variate Feature Selection*. Univariate feature selection approaches are computationally efficient, but they eliminate features owing to a lack of interaction between features that, when combined, may have offered important information regarding classification [104, 105]. When evaluating the performance of features, multi-variate takes into account the interdependencies between them. “Linear multi-variate” employs linear classifiers made up of a subset of features, with the score of feature subsets being calculated based on classification performance. Nonlinear multi-variate, on the other hand, use nonlinear classifiers to complete the task.
  The following are the most often used *linear multi-variate wrapper* approaches in in-text classification:
 
*Recursive Feature Elimination*. It is a recursive strategy that ranks features according to a key measure. During each cycle, the significance of features is assessed, and less relevant features are removed. To design ranking, the opposite process is utilized, in which features are rejected. From this rating, this technique extracts the top-*N* features [106]. This is a greedy optimization that seeks the highest performing feature subset.
 
*Forward/Backward Stepwise Selection*. It is an iterative procedure that begins with the examination of each feature and picks the one that produces the best performing model, based on some predetermined criteria (like prediction accuracy). The next step is to examine every potential combination of that selected feature and the following feature, and if it improves the model, the second feature is chosen. The model continuously appends the list of features that best improve the model's performance in each iteration until the requisite features subset is picked. In the backward feature selection approach, the method starts with the whole collection of features and discards the least relevant feature in each iteration, improving the method's speed. This method is repeated until no improvement is shown when features are removed, and the best subset of features is found. In comparison to other techniques, the researcher developed a rapid forward selection methodology for picking the optimal subset of features that required less computing work [107].
  The *Genetic Algorithm* uses a feature set to generate a better subset of features that are free of noise. At each step, a new subset is formed by picking individual features in the correct sequence and merging those using natural genetics procedures. The result is cross-validated variance divided by the percentage of right predictions. The end outcome may be mutated. This procedure aids in the creation of a feature set of individual features that are more appropriate for the model than the initial feature set. The chaotic genetic algorithm was designed to simplify the feature selection procedure and improve the classification technique's accuracy [108, 109].
  The commonly preferred *nonlinear multi-variate wrapper* methods in the text classification are discussed as follows:
 
*Nonlinear kernel multiplicative updates* entail iteratively training a classifier and rescaling the feature set by multiplying it by a scaling factor that lowers the value of less impacted features. Nonlinear techniques can outperform linear algorithms by selecting a subset of features [110].
 
*Relief* is based on instance-based learning. Each feature receives a value ranging from –1 to +1 based on how well it matches the desired label. The algorithm's scope is binary-classification-compatible [111, 112].

*(3) Embedded Methods*. In terms of computing, “embedded methods” outperform wrappers, but they conduct selection features as a subpart of the learning methodology, which is primarily exclusive to the learning model and may not function with any other classifier.

The commonly preferred embedded methods in the text classification are discussed as follows:

In social sciences, the *LASSO* method is generally used [113]. To alleviate the dimensionality problem, it penalizes features with large coefficients by inserting a penalty during the log-likelihood maximization procedure. By picking a correct weight and reducing dimensionality, LASSO assigns zero to some coefficients. When there is a strong correlation between some features, it creates a difficulty [114].

*Ridge Regression* lowers the complexity of a model by reducing coefficients while keeping all of its features. The issue with ridge regression is that features are retained. If the feature collection is huge, the problem remains complicated [115].

*Elastic Net* calculates a penalty that is a mix of LASSO and ridge penalties. The elastic net penalty may be readily handled to give LASSO or ridge penalties extra power. It has a grouping effect, with high correlation features tending to be in or out of the feature subset. It incorporates both L1 and L2 regularization techniques (LASSO and ridge). By fine-tuning the settings, it aids in the implementation of both strategies [116].

#### 2.4.2. Text Feature Extraction Methods

After selecting the features and representing *N* documents by *d*-dimensional features *vectors*{*X*_1_, *X*_2_,…., *X*_*N*_}. Sometimes original terms in the form of features may not be optimal dimensions for text document representation. The text feature extraction methods try to solve these problems by creating new feature space *Y* or artificial terms [117]. It requires (a) a method to convert old terms to new terms and (b) a method to convert document representation from old to new. A popular example commonly used for this purpose is principal component analysis (PCA) in which a feature set Yi is selected in a manner that the variance of the original feature vectors is maximized in the direction of new feature vectors. This is done by computing eigenvectors of the covariance matrix of the original vectors. The drawback of the PCA is the time it takes to evaluate eigenvalue decomposition to compute the principal component for each class/label when applied to a large data set. This is overcome by the researcher by using the power factorization method (PFM) to find a fixed quantity of eigenvectors from a data set [118]. Another commonly preferred method for feature extraction is latent semantic indexing (LSI) that uses singular value decomposition of the term correlation matrix computed from a large collection of text documents. This technique is used to address the problem of deriving from the use of synonymous and polysemous words as dimensions of the text document representation. But the disadvantage is to compute the correct number of latent components that proves computationally expensive. Another method that helps for optimal discrimination of data is linear discriminant analysis (LDA) [99]. It identifies the linear collection of features that bestexplain the data. It tries to find the model that can explicitly differentiate between the classes of data. Latent Dirichlet allocation is another method that explains that each text document is a mixture of latent topics and each word in that document is attributable to one of the topics of that document. This is most preferred for topic modeling [119]. This is a generative probabilistic model.

A newer approach is a simplified version of stochastic neighbor embedding that creates much better visuals by eliminating the potential to cluster points together in the map's centers known as t-SNE. It visualizes high-dimensional data by assigning a two- or three-dimensional map to each data point. When it comes to constructing a single map that displays structures of several sizes, t-SNE outperforms previous approaches. On almost all of the data sets, the analysis shows that t-SNE produces visuals that are much superior to those produced by the other approaches [120]. Furthermore, the authors provide a technique UMAP (uniform manifold approximation and projection) that is comparable to t-SNE in terms of visualization quality and, in certain ways, retains more of the global structure while providing better run time efficiency [121]. It is based on Laplacian eigenmaps as a mathematical foundation. Umap can scale to far bigger data sets than t-SNE. It is a general-purpose dimension reduction strategy for machine learning since it has no computational constraints on embedding dimensions. The approach is used in the study to evaluate the uniqueness of subjects, important phrases and features, information dissemination speed, and network behaviors for COVID-19 tweets and analysis [122]. To increase the detection of relevant themes in the corpus and analyze the quality of created topics, they use UMAP, which finds distinctive clustering behavior of separate topics [120]. Another study used UMAP to depict the matching word vector spaces of a pretrained language model using LaTeX mathematical formulations. In the LaTeX formula domain, they develop a state-of-the-art BERT-based text classification model augmented by unlabeled data (UL-BERT) [123].

### 2.5. Classifiers for Classification Task

The classifier is trained based on the selected features from the text documents. The selection of appropriate features in feature space decides the performance of learning models. Machines understand numbers more easily than texts as input. So texts as tokens are required to be converted into numbers (vectorization) for most of the learning algorithms. Vectors are combined to originate vector space to apply statistical methods for checking document relatedness. Each algorithm offers a different document representation for text classification. Researchers offer several classification methods that work on the vector representation of texts. The basic assumption for vector representation of texts is known as the contiguity hypothesis. It states that text documents belonging to the same class develop a contiguous region and regions of different classes do not overlap. The relatedness of the documents can be evaluated on 2D space based on cosine similarity or Euclidean distance.

The authors have presented naïve Bayes, a linear classifier, approach to vectorize the text documents according to probability distribution with two commonly used models: multi-variate Bernoulli event and multi-nomial event; the features with the highest probability were chosen to reduce the dimensionality [124].

(11) $$\begin{array}{c} PC_{k}|d_{i}=Pd_{i}|C_{k}*\frac{P\left(C_{k}\right)}{P\left(d_{i}\right)}. \end{array}$$

The output of the classifier is the probability of the text document *d*_*i*_ is belonging to each class *C*_*k*_, and it is a vector of *C* elements. In a way of text classification scenario, we could compute *Pd*_*i*_*|C*_*k*_ using bag-of-words as follows:

(12) $$\begin{array}{c} Pd_{i}|C_{k}=P\left(BoW\left(d_{i}\right)|C_{k}\right)=Pw_{1,i}w_{2,i},,,w_{\left|V\right|,t}|C_{k}. \end{array}$$

The problem can be reduced to compute the probability of each word *w*_*j*,*i*_ in class *C*_*k*_ as follows:

(13) $$\begin{array}{c} Pd_{i}|C_{k}=\prod_{j}^{\left|V\right|}Pw_{j,i}|C_{k}. \end{array}$$

The naïve Bayes has a high bias for a nonlinear problem because it can model one type of class, that is, a linear hyperplane. The bias is the statistical method of evaluating the performance of a classifier that how accurately a classifier classifies the texts into the correct class with less error. The learning task is the last activity in classification, but reducing the feature dimensions is more concerned with the efficiency of the classifier model. The possibility of salient feature reduction caused by using classifiers can be overcome by using the model SVM that identifies the best decision-boundary between feature vectors of the document with their categories [125]. The SVM entails an optimal classifier that guarantees the lowest classification error. The SVM computes a hyperplane that falls between the positive and negative example of the training set.

(14) $$\begin{array}{c} D=\left\{\left(x_{i},y_{i}\right)\left|x_{i}\in R^{D},y_{i}\in \left\{-1,1\right\}\right|\right\}i{=}_{i}^{n}, \end{array}$$

where the minus sign represents the negative hyperplane, the positive sign points to the positive hyperplane, *i* ranges from 1 to *L* (training examples), *(x*_*i*_,*y*_*i*_) represents feature vectors of each document, *R*^*D*^ is a vector space having a dimension of *D*. and *D* is evaluated to +1 and −1 for positive and negative hyperplanes, respectively.

The naïve Bayes itself results in the best classification model if it is trained on a high volume of data. However, feature reduction remains an issue. So naïve Bayes is used as a prestep to SVM that converts text documents into vectors before the classification task starts. This resulted in improving the whole system while spending quite an appropriate classification time by reducing to low-dimensional space. But, in certain cases, the majority of features are redundant with each other; the author has presented a divergence-based feature selection method without relying on a complex dependence model where the maximum marginal relevance-based feature selection was outperformed by the SVM [126]. The paper has suggested the need for novel criteria to measure the relevance and novelty of features separately and provided the linear combination as the metric.

The studies have mentioned the KNN, a nonlinear classifier, where the algorithm classifies the document by moving through all the training documents that are like that document. The KNN model frames the documents in the Euclidean space as points so that the distance between two-point *u* and *v* can be calculated as follows:

(15) $$\begin{array}{c} D{\left(u,v\right)}^{2}=u-v^{2}={\left(u-v\right)}^{T}\left(u-v\right)=\sum_{i-1}^{d}{\left(u_{i-}v_{i}\right)}^{2}. \end{array}$$

The classifier finds the *K*-value that is the factor that represents a collection of documents from all the documents closest to the selected document in that space [127]. If there are too many features, KNN may not operate effectively. As a result, dimensionality reduction techniques such as feature selection and principal component analysis may be used.

(16) $$\begin{array}{c} \hat{y}\left(x\right)=y_{n^{*}}\text{ where }n^{*}=\text{arg}\underset{n\in D}{\min}\text{dist}\left(x,x_{n}\right). \end{array}$$

The study mentions that using KNN increases the overhead to calculate the *K*-value of all the documents with all other training documents with the largest similarity or closet to the selected document. Also, the variation in the number of training sample documents in different categories leads to a decline in accuracy. Due to the high variance and complex regions between classes, it becomes sensitive to noise documents. Sometimes, the document tends to misclassify if it occurs very relevant to a noise document in the training set and sometimes accurately classified if there is no presence of noise documents in the training set close to them. This ends up in high variance from the training set to the training set. High variance leads to overfitting. The goal of finding a good learning classifier is to decrease the learning error. Learning error is calculated from bias and variance or bias-variance trade-off. The traditional algorithms possess some limitations that attract the researchers to improve the efficiency by (a) reducing the computational overheads by establishing low-dimensional space, (b) speeding up the computational capacity of finding nearest neighbors in KNN or locating decision boundaries in SVM, and (c) increasing efficiency by not compromising accuracy [128]. The model is chosen that optimizes the fit to the training data.

(17) $$\begin{array}{c} H^{*}=\text{arg}\underset{H}{\max}\text{fit}\left(H|D\right). \end{array}$$

The supervised learning classification algorithms such as naïve Bayes, SVM, and KNN use a bag of words to design a classifier. None of these methods take the order of words into consideration that can lead to the loss of some valuable information. In natural-language-based problems, the order of the words, for example, multi-words, has a meaning (like names of organization or person) that is not considered by the learning models trained on individual words of the texts. The algorithm *n*-grams consider the sequence of *n*-adjacent words from the selected text phrases. The *n*-grams behave like the individual word's representation as feature vectors. The value of *n* may range from 1 to the upper value [129]. This proves very beneficial in short text documents where the number of *n*-grams is less in number [89]. The author proposes another representation for character *n*-grams to introduce the enhancement of the skip-gram model, which considers subword into account. It considers the word morphology while training the model, and words are represented by the sum of its character *n*-gram [55].

Many researchers have used a decision-tree-based algorithm (decision support tool) that represents a tree-structure-based graph of decisions [130]. The commonly used decision tree algorithms are ID3, C4.5, and C5. The algorithm presents each intermediate node (labeled as terms and branches represent weight) that can split into subtrees and ends at leaf nodes (represents the class/label/outcome of the problem). Decision tree structures are rule-based solutions. A rule can be designed by forming a conjunct of every test that occurs on the path between the root node and the leaf node of the tree. The rules are formed after traversing every path from a root to the leaf node. Once the decision tree and rule are framed, it helps assign the class/label for a new case [129, 131]. It was evaluated in the study that decision trees result better than naïve Bayes in terms of accuracy but a little worse than KNN methods [132]. As a result, the authors introduce the boosting data classification approach. The boosting algorithm is a method for combining many “poor” classifiers into a single, powerful classifier. The boosting technique is used on decision trees in the study, and the boosted decision tree performs better than an artificial neural network [133]. It is expected to find widespread use in a variety of fields, particularly text classification. Gradient tree boosting is another boosting strategy that builds an additive regression model using decision trees as the weak learner. Trees in stochastic gradient boosting are trained on a randomly selected portion of the training data and are less prone to overfitting than shallow decision trees [134].

Neural networks or deep learning systems use several processing layers to learn hierarchical data representations and have reached state-of-the-art outcomes in most domains. In the sense of natural language processing (NLP), several model designs and approaches have recently progressed. In areas such as computer vision and pattern recognition, deep learning architectures and algorithms have also made remarkable progress. Recent NLP research is now primarily focused on the application of new deep learning approaches, continuing this development. In areas such as computer vision and pattern recognition, deep learning architectures and algorithms have also made remarkable progress. Recent NLP research is now primarily focused on the application of new deep learning approaches, continuing this development. The performance of word embedding and deep learning strategies referred to in the following section is driving this development.

In the late 90s, the researcher found an application of nonlinear neural networks to text classification or topic modeling [135]. In this model, a three-layered neural network was designed to learn a nonlinear mapping from training documents to each class/label. Later on, the researcher proposes convolutional neural network (CNN) for text classification by considering the order of words in the phrases, and this outperforms SVM in terms of error rate [136]. CNN uses the vector representation of text data considering the order of words. Each word is considered a pixel, and the document is treated as an image. Then the image is taken into |D| × 1 pixels, and each pixel represent a word as a |V| dimensional vector. For instance, vocabulary *V* = {“classification”, “course”, “I”, “love,” “NLP',” “text”}, and words are taken as a dimension of vectors in alphabetical order. And document *D* = “I love NLP course”. Then the document vector would be *X* = [0010000 | 000100 | 00001 | 010000].

The researcher mentions that to reduce the dimensionality of vector space, the vocabulary size must be kept low. Also, the *n*-gram algorithm ignores the fact that some *n*-grams share the constituent words; this is overcome by CNN that learns the embedding of text regions by providing CNN with the constituent words as input, and this technique provides higher accuracy. To construct an informative latent semantic representation of the sentence for downstream activities, CNNs have the potential to extract salient *n*-gram features from the input sentence. The author proposes a three-way enhanced CNN for classification for sentiment analysis, where decisions are divided into three parts accept, reject, and delay. The instances in boundary regions that are neither in accept nor reject are reclassified by another classification model. This guarantees the enhancement in CNN to deal with boundary regions in a better way, resulting in model 3W–CNN [137]. Another study has shown the application of CNN in document modeling for personality detection based on text in the context of sentiment analysis [34]. Overall, in contextual windows, CNNs are highly successful in mining semantic hints. They are very data-heavy models, though. They have a huge range of parameters that are trainable and need tremendous training data. This raises a concern as data shortage happens. Another unresolved concern with CNNs is their failure to model contextual long-distance data and maintain sequential order in their representations [138].

Deep neural networks are difficult to train on data; it requires a lot of resources to get high performance. The feed-forward neural network commonly known as multi-layer perceptron (MLP) is the most preferred technique in classification problems. In a feed-forward neural network, the information travels in one direction from the input layer to hidden layers and then followed by the output layer. They have no memory of the input received previously so lack in predicting what comes next. To overcome this, RNN (recurrent neural network) is preferred where information moves through the loop. In this paragraph, we discuss the fundamental characteristics that have favored the popularization of RNNs in a variety of NLP tasks. Since an RNN performs sequential processing in sequence by modeling units, it may have the ability to generate the intrinsic sequential structure present in language, where characters, words, or even phrases are units. Based on the previous words in the sentence, words in a language establish their semantic meaning. The disparity in interpretation between “computer” and “computer vision” is a clear example that states this. RNNs are perfect for language and related sequence modeling activities to predict certain context dependencies, which turned out to be a clear incentive for researchers to use RNNs over CNNs in these fields.

RNNs were originally three-layer networks in the NLP sense [139]. While deciding on the current input layer, it considers what it has learned from the previous inputs. Basically, in the architecture of simple RNN, the hidden units create internal representations for the input patterns and recode these patterns in feed-forward networks using hidden units and a learning algorithm in a way that allows the network to generate the appropriate output for a given input. Typically, the hidden state of the RNN is assumed to be the most important feature. It can be regarded as the memory portion of the network that accumulates data from other steps.

The formula of the current state in RNN can be written as mentioned below. A nonlinear transformation such as tanh, or ReLU, is taken to be the function *f*.

(18) $$\begin{array}{c} h_{t}=f\left(h_{t-1},x_{t}\right), \end{array}$$

where *h*_*t*_ is the new state, *h*_*t–1*_ is the previous state, and *X*_*t*_ is the input at time *t*.

The tanh function is commonly used as an activation function. The weights can be defined as the matrix *W*_*hh*_, and input is defined by the matrix *W*_*xh*_:

(19) $$\begin{array}{c} h_{t}=\text{tan }h\left(W_{hh}h_{t-1}+W_{xh}X_{t}\right). \end{array}$$

The output can be calculated during test time as follows:

(20) $$\begin{array}{c} y_{t}=W_{hy}h_{t}. \end{array}$$

The output is then compared to the actual output, and then the error value is computed. The network learns by backpropagating the error through the network to update the weights. But usual RNN has a short-term memory. These basic RNN networks suffer from the issue of vanishing gradient, which makes it very difficult to understand and adjust the parameters of the previous layers in the network. It is used in combination with LSTM, which has long-term memory, and it gives an extension to the memory of the usual RNN. Over the basic RNN, LSTM has additional “forget” gates, allowing the error to backpropagate over an infinite amount of time steps. Comprising three gates: input, forget, and output gates, taking a combination of these three gates, it determines the hidden state [22]. The applications based on RNN and LSTM have been used in solving many NLP problems due to their capacity of capturing complex patterns within the text [140]. It has also been used in sequence labeling tasks in POS (part of speech) activity. It is preferably used in topic modeling for fake news [141, 142], sentiment analysis [143, 144], and negative speech detection on social media. More recently, authors have suggested another type of recurrent unit, which they refer to as a gated recurrent unit (GRU) [145]. It has been shown that RNNs employing any of these recurrent units perform well in tasks (such as machine translation, speech recognition, or depending parsing in text documents for NER) requiring long-term dependency capture. The application of gated RNN is not limited to the mentioned tasks, but it can be applied to different NLP challenges [146]. GRU is a form of recurrent neural network (RNN) that can process sequential data using its recurrent architecture. The fundamental issue in text classification is how to improve classification accuracy, and the sparsity of data, as well as semantics sensitivity to context, frequently impedes text classification performance. The study introduces a unified framework to evaluate the impacts of word embedding and the gated recurrent unit (GRU) for text classification to overcome the flaw [147].

Recurrent neural networks, in particular long short-term memory [22], and gated recurrent neural networks [62] are firmly known as state-of-the-art approaches in sequence modeling. Within training examples, the inherently sequential nature of recurring models prevents parallelization, which becomes important at longer sequence lengths, as memory limitations restrict batching through examples. Recent work, through factorization tricks [148] and conditional computation [149], has achieved substantial improvements in computational efficiency while also improving model output in the case of the latter. The authors have presented two simple ways of reducing the number of parameters and speeding up the training of large long short-term memory networks: the first is the “matrix factorization by design” of the LSTM matrix into two smaller matrices, and the second is the division into separate classes of the LSTM matrix, its inputs, and its states. However, the essential restriction of sequential computation persists.

Attention mechanisms have become an integral part of sequence modeling in different applications, allowing dependencies to be modeled regardless of their gap in the input or output sequences. The following paragraph examines some of the most prominent models of attention that have created new state-of-the-art tasks for text classification. The study's conclusion was largely focused on text classification experiments that could not be extended to many other NLP tasks [150]. The authors mentioned that attention offers a reliable explanation for model predictions, expecting these properties to hold (a) attention weights should align with feature-relevant measurements (e.g., gradient-based measurements) and (b) alternative (or counterfactual) weight configurations should result in corresponding prediction changes. They stated that in the sense of text classification, neither property is consistently observed by a Bi-LSTM with a standard attention mechanism. The layers of attention specifically weigh the representations of the input elements; it is also often believed that attention can be used to classify information that was considered relevant by models. In another study, the authors evaluate if that assumption holds by modifying weights of attention in already trained text classification methods and examining the resulting variations in their predictions. Although experimenting with text classification, the authors note several cases in which higher attention weights correlate with a greater impact on model predictions, they also notice several cases this does not hold, that is, where gradient-based attention weight rankings predict their effects better than their magnitudes [151]. In contrast to the current work on interpretability, the authors in another research reported that they examined the attention mechanism on a more diverse set of NLP tasks that included text classification, pairwise text classification, and tasks for generating text such as neural machine translation [152].

Although the hidden vectors represented by an attention model through encoding can be interpreted as internal memory entries for the model, memory-augmented networks integrate neural networks with an external memory type that the model could learn from it and respond to. For text classification, the study proposes a memory-augmented neural network called the neural semantic encoder [153]. In another research, the authors introduce neural network architecture, the dynamic memory network (DMN), which processes input sequences and questions, shapes episodic memories, and generates appropriate responses. The model possesses an iterative mechanism of attention that allows the model to condition its attention on the inputs and outcomes of previous iterations. In a hierarchical recurrent sequence model, these outcomes are then reasoned over to produce answers [154]. The authors also stated that it is possible to train the DMN end-to-end and obtain state-of-the-art text classification results using the Stanford Sentiment Treebank data collection.

Sequential processing of text is one of the system inefficiencies experienced by RNNs. Transformers address this constraint by applying self-attention to measure an “attention ranking” in parallel for each word in a phrase or document to model the impact each word has on another. Because of this feature, Transformers allow for far more parallelization than CNNs and RNNs, allowing very large models to be efficiently trained on large quantities of data on GPU stacks [155]. The Transformer architecture is especially suitable for pretraining large text corpus, leading to significant accuracy improvements in downstream tasks, including text classification [69]. They propose a novel Transformer-XL neural architecture that allows learning dependence to interrupt temporal coherence beyond a fixed length. We have seen the emergence of several large-scale transformer-based pretrained language models in the current scenario. As stated in Section 2.2.2, these Transformers are pretrained to learn contextual text representations in much greater volumes of text corpora by predicting terms that are trained on their context. These pretrained models were fine-tuned using task-specific tags and in many subsequent NLP tasks, especially text classification produced new state-of-the-art. Fine-tuning is supervised learning, while pretraining is unsupervised.

The authors design the largest model, OpenGPT, 1.5 B parameter Transformer that ensures state-of-the-art results and comprises 12 layers of Transformer frames, each composed of a masked multi-head attention unit, followed by a standardization layer and a forward feed layer in place. With the addition of task-specific linear classifiers and fine-tuning with task-specific tags, OpenGPT can be extended to the text classification. Unlike OpenGPT that predicts words based on previous predictions, there is another model that comes into use, that is, BERT, intended to pretrain deep bidirectional representations from the unlabeled text by conditioning in all layers on both the left and right context together [71]. For text classification, BERT variants have been fine-tuned [156]. ALBERT decreases memory usage and improves BERT's training speed [157]. Another variant of BERT, SpanBERT [158], is a pretraining method designed to accurately represent and forecast spans of text. It improves BERT by (1) masking consecutive random spans, rather than random tokens, and (2) training the span boundary representations to estimate the entire content of the masked span without relying on the individual token representations within it. Deep learning provides a way to manage massive volumes of processing and data with next to no engineering by hand [23]. Unsupervised learning has had a catalytic impact on the growing interest in deep learning, but the contributions of solely supervised learning have since been overshadowed. In the longer term, we expect unsupervised learning to become even more significant.

## 3. Evaluation

We have discussed several supervised and unsupervised text classification methods based on machine and deep learning models so far; however, the shortage of uniform data collection procedures is a big issue when testing text classification techniques. Even if there is a standard collection method available, it can generate differences in model results by simply selecting different training and test sets [146]. Moreover, to compare various performance measures used during separate tests, there may be another difficulty related to process evaluation. In general, performance metrics assess attributes of the performance of the classification task and therefore do not necessarily present similar information. Although the underlying mechanics of various measurement metrics vary, it is important to consider precisely what each of these metrics describes and what kind of data they are attempting to express for comparability. Some examples of such performance measures include precision, recall, accuracy, microaverage, macroaverage, and F-measure. These calculations are based on a “confusion matrix” composed of true positive, false positive, false negative, and true negative [47]. Accuracy is considered the fraction of accurate predictions in overall predictions. The fraction of known positives accurately estimated is referred to as recall. The fraction of positives accurately estimated for all positives is called precision.

Another technique for evaluating how well our machine learning models perform on unknown data is cross-validation. If we expose the model to entirely new, previously unknown data, it may not be as accurate in predicting and may fail to generalize over the new data. Overfitting is the term for this issue. Because it is unable to discover patterns, the model does not always train effectively on the training set. It would not do well on the test set in this situation. Underfitting is the term for this issue. We employ cross-validation to solve overfitting issues. A cross-validation is a resampling approach in which the data set is split into two parts: training data and test data. The model is trained using training data, and the model is predicted using test data that has yet to be observed. If the model performs well on the test data and has a high level of accuracy, it has not overfitted the training data and may be used to forecast. *K*-fold cross-validation is the most basic type of cross-validation. Other types of cross-validation include variations on *k*-fold cross-validation or entail repeating *k*-fold cross-validation rounds. The data is initially partitioned into *k* equally sized segments or folds in *k*-fold cross-validation. Following that, *k* iterations of training and validation are undertaken, with each iteration holding out a different fold of the data for validation and the remaining *k*-1 folds being employed for learning [147]. For each of the *k* “folds”, the following approach is used:

- The folds are used as training data to train a model
- The generated model is validated using the remaining data (i.e., it is used as a test set to compute a performance measure such as accuracy)

In cases where there is uncertainty, entropy is especially appealing as a predictor of classification quality. It denotes how well the class membership probabilities are distributed throughout the specified classes. Its usefulness as a predictor of classification accuracy is predicated on the notion that in an accurate classification, each sentence has a high likelihood of belonging to just one class [159]. Cross-entropy loss is a key indicator for evaluating the performance of a classification issue. The prediction is a probability vector, which means that it reflects the anticipated probabilities of all classes, which add up to one. In a neural network, this is commonly accomplished by activating the final layer with a softmax function, but anything goes, it just has to be a probability vector. Maximum entropy is used in our text classification scenario to estimate the conditional distribution of a class label given a document. A collection of word-count characteristics represents a document. On a class-by-class basis, the labeled training data is utilized to estimate the anticipated value of these word counts. Iterative scaling is improved to obtain a text classifier with an exponential shape that is compatible with the limitations of the labeled data [160].

## 4. Comparative Analysis

The following section summarizes the benefits and limitations of feature extraction, feature selection methods, and supervised and unsupervised machine and deep learning models used for a text classification task.

### 4.1. Comparative Analysis of Standard Text Representation or Feature Extraction Methods

The two major feature extraction methods are highlighted: weighted words and embedding of words. By considering their frequency and co-occurrence details, word embedding approaches learn through sequences of words. These strategies are also unsupervised models to create word vectors. In comparison, the properties of weighted terms are based on counting words in documents and can be used as a basic word representation ranking scheme. Each approach poses specific constraints. Weighted words explicitly quantify text similarities from the word-count space, which enhances the computational time for large vocabulary. Although counting unique terms offers independent confirmation of similarity, semantic comparisons between words are not taken into consideration [148]. Word embedding techniques solve this challenge but are constrained by the need for a large corpus of text data sets to train [21]. Therefore, researchers tend to use vectors with pretrained word embedding [149].  Table 1 presents the advantages and limitations of each technique of text representation or feature extraction.

### 4.2. Comparative Analysis of Standard Feature Selection Methods

Some studies have preferred Fisher's linear discriminant for the support-vector-based feature ranking model [99, 100]. The author has mentioned that the filter-based method provides distinguish feature selector that has further selected distinctive features that possess term characteristics during the elimination of uninformative ones [92]. It offered performance by decreasing processing time and increasing classification accuracy. Another technique in the wrapper method has gained popularity fast forward selection technique for selecting the best subset of a feature that demanded less computational effort as compared to other methods [107]. In the genetic algorithmic approach, a chaos genetic algorithm was proposed to simplify the feature selection method and obtained higher accuracy of classification technique [108, 109, 161]. Embedded methods were preferred over wrappers in many studies. Researchers mentioned that embedded methods have performed better than wrapper computationally, but these algorithms perform selection features as a subpart of the learning technique. Furthermore, these were followed by hybrid feature selection approaches where both filter and wrapper methods were combined, and these approaches were proven more computationally effective than the performance of a single selection technique. It was observed by the researchers that sometimes original features may not be optimal dimensions for text document representation. They provided text feature extraction methods to solve the problem by creating new feature space or artificial terms [117].  Table 2 presents the benefits and limitations of feature selection methods.

### 4.3. Comparison of State-of-the-Art Machine and Deep Learning Models for Text Classification

The performance of the classifier depends on the selection of feature selection and extraction method. The supervised and unsupervised machine learning techniques have offered a variety of classifiers that performed well in a variety of domain-specific classification problems. The following Table 3 presents their pros and cons. Recent studies have focused on deep learning or neural network-based classifiers such as CNN, RNN, and RNN with LSTM that have shown better results than conventional algorithms such as SVM and KNN in solving a different range of problems. RNN and LSTM have been used in many NLP applications due to their capacity of capturing complex patterns within the text [151]. It has also been used in sequence labeling tasks in POS (part of speech) activity. But it has offered a lot of future scope in resolving complexities involved in the backpropagation technique used in RNN and making the learning model cheaper and faster [104].

## 5. Research Gap and Future Direction

From this review of existing studies, we identified that there exist certain gaps, which we can plan to fill in the future. While labeling unstructured text data manually, it takes a lot of time to understand the data to categorize it. Also, it needs specialists for understanding domain-specific data. In such scenarios, the machine learning algorithms do not produce the expected accuracy [12]. Extraction of meaning or finding semantic relations between words of unstructured data is a complex task, which has been tried by several authors using NLP techniques for years [24, 162]. However, these methods prove inefficient when pursuing building a high-quality classification system. It offers opportunities to design semisupervised machine learning models to label some parts of training data manually, and the rest data can be trained using machine learning algorithms [163].

Apart from conventional methods used for representing data sets for extracting patterns from the text data using vector representation based on word-embedding and paragraph-embedding [38, 58], the authors have presented deep neural network models for NLP-based applications using character-embedding [164], unsupervised techniques based on transfer learning, Bert, with fine-tuning with domain-specific data [156]. However, these representations and globally available representations cannot be generalized to unseen texts, which are very specific to a particular domain [59]. It offers opportunities to design methodology for extending the vocabulary of existing representations for specific domains.

The deep learning algorithms are proven good in decision-making for NLP-based applications, and these models cannot handle symbols directly [165]. Also, the computational cost of training such algorithms is very high. It offers scope for designing deep neural network-based architecture, which can be inputted with linguistic knowledge, lexical knowledge, and word knowledge from different domains.

## 6. Conclusions

In this paper, we have provided a detailed review of the complete process of the text classification system. This paper covered various algorithms or methods used in subtasks of classification. It has presented the techniques for data collection from several online sources. The documents were represented with basic techniques and followed by recent research in document representation for different areas of machine learning and natural language processing. To provide suitable and fast classifiers the higher dimensional space of data was reduced to a lower space using feature selection and feature extraction methods. Different algorithms perform differently depending on the domain-specific data collections and to train machine learning text classifiers. The authors have used these algorithms based on the problem statement, and none of the algorithms has proven perfect for all types of problems and data dimensionality.

It is observed that in recent years, some studies focused on new applications of text classification such as multi-label classification [166, 167], and hierarchical classification [168, 169] in the field of natural language processing or machine translation and medical sciences, respectively. The neural network-based algorithms are commonly used in NLP-based problems [136, 137, 142]. However, these algorithms mainly focus on generic data. Moreover, CNN is preferably used for image processing, and RNN with LSTM is preferred for time-series problems. Initially, these algorithms were not applied to text data, but in recent years, CNN with character-embedding is being used for document representation and feature selection in text documents [58]. The transformers-based unsupervised models also overcome the issue of RNN and LSTM, but these techniques are proven computationally expensive. With the advancement of deep neural networks, we may find in the future that these deep neural networks will be applied efficiently in the automatic monitoring of web-based text data and classifying unseen data into automated labels [7].

## Figures and Tables

**Table 1:** Benefits and limitations of text representation or feature extraction methods.

Method	Benefits	Limitations
Bag-of-words	Works well with unseen words and is easy to implement as it is based on the most frequent terms in a document	Does not cover the syntactic and semantic relation of the words, common words impact classification
TF-IDF	In like bag-of-words approach, common words are excluded due to IDF so does not impact the result	Does not cover the syntactic and semantic relation of the words
Word2vec	Covers the syntactic and semantic relation of the words in the text	Does not cover the words' polysemy
GloVe	As the same as word2vec but performs better, eliminates common words, trained on a large corpus	Does not cover the words' polysemy and does not work well for unseen words
Context-aware representation	Covers the context or meaning of the words in the text	Huge memory is required for storage and does not work well for unseen words

**Table 2:** Benefits and limitations of feature selection methods.

Method		Benefits	Limitations
Univariate filter method	Information gain	Results into the relevance of an attribute or feature	Biased towards multi-valued attributes and overfitting
	Chi-square	Reduces training time and avoids overfitting	Highly sensitive to sample size
	Fishers' score	Evaluates features individually to reduce the feature set	Does not handle features redundancy
	Pearson's correlation coefficient	Is simplest and fast and measures the linear correlation between features	It is only sensitive to a linear relationship
	Variance threshold	Removes features with variance below a certain cutoff	Does not consider the relationship with the target variable

Multi-variate filter method	mRMR (minimal redundancy maximum relevance)	Measures the nonlinear relationship between feature and target variable and provides low error accuracies	Features may be mutually as dissimilar to each other as possible
	Multi-variate relative discriminative criterion	Best determines the contribution of individual features to the underlying dimensions	Does not fit for a small sample size

Linear multi-variate wrapper method	Recursive feature elimination	Considers high-quality top-*N* features and removes weakest features	Computationally expensive and correlation of features not considered
	Forward/backward stepwise selection	Is computationally efficient and greedy optimization	Sometimes impossible to find features with no correlation between them
	Genetic algorithm	Accommodates data set with a large number of features and knowledge about a problem not required	Stochastic nature and computationally expensive

Nonlinear multi-variate wrapper methods	Nonlinear kernel multiplicative	De-emphasizes the least useful features by multiplying features with a scaling factor	The complexity of kernel computation and multiplication
	Relief	Is feasible for binary classification, based on nearest neighbor instance pairs and is noise-tolerant	Does not evaluate boundaries between redundant features, not suitable for the low number of training data sets

Embedded methods	LASSO	L1 regularization reduces overfitting, and it can be applied when features are even more than the data	Random selection when features are highly correlated
	Ridge regression	L2 regularization is preferred over L1 when features are highly correlated	Reduction of features is a challenge
	Elastic net	Is better than L1 and L2 for dealing with highly correlated features, is flexible, and solves optimization problems	High computational cost

**Table 3:** Benefits and limitations of the machine and deep learning model.

Model	Benefits	Limitations
Naïve Bayes	It needs less training data; probabilistic approach handles continuous and discrete data; and it is not sensitive to irrelevant features, easily updatable	Data scarcity can lead to loss of accuracy because it is based on assumption that any two features are independent given the output class.

SVM	It is possible to apply to unstructured data also such as text, images, and so on; kernel provides strength to the algorithm and can work for high-dimensional data	It needs long training time on large data sets and is difficult to choose good kernel function, and choosing key parameters varies from problem to problem.

KNN	It can be implemented for classification and regression problems and produces the best results if large training data is available or even noisy training data, preferred for multi-class problems	Cost is high for computing distance for each instance; finding attributes for distance-based learning is quite a difficult task; imbalanced data causes problems; and no treatment is required for missing value.

Decision tree	It reduces ambiguity in decision-making; implicitly performs feature selection, easy representation, and interpretation; and requires fewer efforts for data preparation	It is unstable due to the effect of changes in data requires changes in the whole structure, is not suitable for continuous values, and causes overfitting problem.

Boosted decision tree	It is highly interpretable and prediction accuracy is improved. It can model feature interactions and execute feature selection on its own. Gradient boosted trees are less prone to overfitting since they are trained on a randomly selected subset of the training data.	These are computationally expensive and frequently need a large number of trees (>1,000), which can take a long time and consume a lot of memory.

Random forest	In contrast to other methods, clusters of decision trees are very easy to train, and the preparation and preprocessing of the input data do not require.	More trees in random forests increase the time complexity in the prediction stage, and high chances of overfitting occur.

CNN	It provides fast predictions, is best suited for a large volume of data, and requires no human efforts for feature design.	Computationally expensive requires a large data set for training.

RNN	It implements feedback model so considers best for time series problems and makes accurate predictions than other ANN models.	Training of model is difficult and takes a long time to find nonlinearity in data, and gradient vanishing problem occurs.

LSTM, Bi-LSTM	Adds short- and long-term memory components into RNN so it considers best for applications that have a sequence and uses for solving NLP problems such as text classification and text generation, and computing speed is high. Bi-LSTM solves the issue of predicting fixed sequence to sequence.	It is expensive and complex due to the backpropagation model, increases the dimensionality of the problem, and makes it harder to find the optimal solution. Since Bi-LSTM has double LSTM cells so it is expensive to implement.

Gated RNN (GRU)	In natural language processing, GRUs learn quicker and perform better than LSTMs on less training data. As it requires fewer training parameters. GRUs are simpler and hence easier to modify and do not need memory units, such as by adding extra gates if the network requires more input.	Slow convergence and limited learning efficiency are still issues with GRU.

Transformer with an attention mechanism	The issue with RNNs and CNNs is that when sentences are too long, they are not able to keep up with context and content. By paying attention to the word that is currently being operated on this limitation was resolved, the attention strategy is an effort to selectively concentrate on a few important items while avoiding those in deep neural networks to execute the same operation, enabling much more parallelization than RNNs and thus reduces training times.	At inference time, it is strongly compute-intensive.

## Data Availability

The data used in this study are publicly available.

## References

[1] Atkins A., Niranjan M., Gerding E. (2018). Financial news predicts stock market volatility better than close price. *The Journal of Finance and Data Science*.

[2] Robinson J., Glean A., Moore W. (2018). How does news impact on the stock prices of green firms in emerging markets?. *Research in International Business and Finance*.

[3] Löffler G., Norden L., Rieber A. (2016). *Salient News and the Stock Market Impact of Tone in Rating Reports*.

[4] Elagamy M. N., Stanier C., Sharp B. Stock market random forest-text mining system mining critical indicators of stock market movements.

[5] Zhang Y., Dang Y., Chen H., Thurmond M., Larson C. (2009). Automatic online news monitoring and classification for syndromic surveillance. *Decision Support Systems*.

[6] West J. C., Clarke D. E., Duffy F. F. (2016). Availability of mental health services prior to health care reform insurance expansions. *Psychiatric Services*.

[7] Ali M., Khalid S., Rana M. I., Azhar F. A probabilistic framework for short text classification.

[8] Romero C., Ventura S. (2007). Educational data mining: a survey from 1995 to 2005. *Expert Systems with Applications*.

[9] Ye Q., Zhang Z., Law R. (2009). Sentiment classification of online reviews to travel destinations by supervised machine learning approaches. *Expert Systems with Applications*.

[10] Thorleuchter D., Van Den Poel D. (2012). Predicting e-commerce company success by mining the text of its publicly-accessible website. *Expert Systems with Applications*.

[11] Yang G., Jan M. A., Rehman A. U., Babar M., Aimal M. M., Verma S. (2020). Interoperability and data storage in Internet of multimedia things: investigating current trends, research challenges and future directions. *IEEE Access*.

[12] Gandomi A., Haider M. (2015). Beyond the hype: big data concepts, methods, and analytics. *International Journal of Information Management*.

[13] Sun Y., Wong A. K. C., Kamel M. S. (2009). Classification of imbalanced data: a review. *International Journal of Pattern Recognition and Artificial Intelligence*.

[14] Manne S., Fatima S. S. (2011). A novel approach for text categorization of unorganized data based with information extraction. *International Journal of Computational Science and Engineering*.

[15] Harish B. S., Guru D. S., Manjunath S. (2010). Representation and classification of text documents: a brief review. *IJCA, Spec Issue Recent Trends Image Process Pattern Recognit*.

[16] Liang Y., Liu Y., Kwong C. K., Lee W. B. (2012). Learning the “Whys”: discovering design rationale using text mining - an algorithm perspective. *Computer-Aided Design*.

[17] Liu B., Li X., Lee W. S., Yu P. S. (2004). Text classification by labeling words. *Artificial Intelligence*.

[18] Zhou D. Y., Bousquet O., Lal T. N., Weston J., Schölkopf B. (2004). Learning with local and global consistency. *Advances in Neural Information Processing Systems*.

[19] Rehman H. U., Rafique R., Nasir M. C. M. (2017). Forecasting CO2 emissions from energy, manufacturing and transport sectors in Pakistan: statistical vs. Machine learning methods. *Mach Learn Methods*.

[20] Kundu N., Rani G., Dhaka V. S. (2021). IoT and interpretable machine learning based framework for disease prediction in pearl millet. *Sensors*.

[21] Bengio Y., Ducharme R., Vincent P. (2001). A neural probabilistic language model. *Advances in Neural Information Processing Systems*.

[22] Hochreiter S., Schmidhuber J. (1997). Long short-term memory. *Neural Computation*.

[23] Lecun Y., Bengio Y., Hinton G. (2015). Deep learning. *Nature*.

[24] Ittoo A., Nguyen L. M., Van Den Bosch A. (2016). Text analytics in industry: challenges, desiderata and trends. *Computers in Industry*.

[25] Da Costa Albuquerque F., Casanova M. A., De Macedo J. A. F., de Carvalho M. T. M., Renso C. A proactive application to monitor truck fleets.

[26] Schmidt S., Schnitzer S., Rensing C. (2016). Text classification based filters for a domain-specific search engine. *Computers in Industry*.

[27] Hussain A., Nazir S., Khan F. (2021). A resource efficient hybrid proxy mobile IPv6 extension for next generation IoT networks. *IEEE Internet of Things Journal*.

[28] Kumar M., Mukherjee P., Verma K., Verma S., Rawat D. B. (2021). Improved deep convolutional neural network based malicious node detection and energy-efficient data transmission in wireless sensor networks. *IEEE Transactions on Network Science and Engineering*.

[29] Rani P., Kavita V. S., Verma S., Nguyen G. N. (2020). Mitigation of black hole and gray hole attack using swarm inspired algorithm with artificial neural network. *IEEE Access*.

[30] Billal B., Fonseca A., Sadat F. Named entity recognition and hashtag decomposition to improve the classification of tweets.

[31] Schumaker R. P., Chen H. (2009). A quantitative stock prediction system based on financial news. *Information Processing & Management*.

[32] Chen Y., Li Z., Nie L., Hu X., Wang X. (2012). Supervised bayesian network model for microblog topic classification.

[33] Xia R., Zong C., Hu X., Cambria E. Feature ensemble plus sample selection: domain adaptation for sentiment classification.

[34] Majumder N., Poria S., Gelbukh A., Cambria E., Cambria E. (2017). Deep learning-based document modeling for personality detection from text. *IEEE Intelligent Systems*.

[35] Cambria E. (2016). Affective computing and sentiment analysis. *IEEE Intelligent Systems*.

[36] Gaur L., Singh G., Solanki A. (2021). Disposition of Youth in Predicting Sustainable Development Goals Using the Neuro-Fuz. *Human-Centric Computing and Information Sciences*.

[37] Pinheiro R. H. W., Cavalcanti G. D. C., Tsang I. R. (2017). Combining dissimilarity spaces for text categorization. *Information Sciences*.

[38] Sabbah T., Selamat A., Selamat M. H. (2017). Modified frequency-based term weighting schemes for text classification. *Applied Soft Computing*.

[39] Dogra V., Verma S., Verma K., Ghosh N., Le U., au D.-N. (2022). A comparative analysis of machine learning models for banking news extraction by multiclass classification with imbalanced datasets of financial news: challenges and solutions. *International Journal of Interactive Multimedia and Artificial Intelligence*.

[40] Zhao Y., Qin B., Liu T. (2015). Creating a fine-grained corpus for Chinese sentiment analysis. *IEEE Intelligent Systems*.

[41] Zhang Y., Jin R., Zhou Z. H. (2010). Understanding bag-of-words model: a statistical framework. *International Journal of Machine Learning and Cybernetics*.

[42] Joseph T., Lev Ratinov Y. B. Word representations: a simple and general method for semi-supervised learning.

[43] Silipo R., Melcher K. (2019). *Text Encoding: A Review*.

[44] Sivic Z., Zisserman Video Google: a text retrieval approach to object matching in videos.

[45] Jing Li-P., Huang H.-K., Shi H.-Bo Improved Feature Selection Approach TFIDF in Text Mining.

[46] Montañés E., Díaz I., Ranilla J., Combarro E., Fernandez J. (2005). Scoring and selecting terms for text categorization. *IEEE Intelligent Systems*.

[47] Li Z., Verma S., Jin M. (2021). Power allocation in massive MIMO-HWSN based on the water-filling algorithm. *Wireless Communications and Mobile Computing*.

[48] Bengio Y., Ducharme R., Pascal V., Christen J. (2003). A neural probabilistic language model. *Journal of Machine Learning Research*.

[49] Xiong Y., Chen S., Qin H. (2020). Distributed representation and one-hot representation fusion with gated network for clinical semantic textual similarity. *BMC Medical Informatics and Decision Making*.

[50] Kowsari K., Jafari Meimandi K., Heidarysafa M., Mendu S., Barnes L., Brown D. (2019). Text classification algorithms: a survey. *Information*.

[51] Mikolov T., Chen K., Corrado G., Dean J. Efficient estimation of word representations in vector space.

[52] Liu B. (2020). Text sentiment analysis based on CBOW model and deep learning in big data environment. *Journal of Ambient Intelligence and Humanized Computing*.

[53] Schwenk H. (2007). Continuous space language models. *Computer Speech & Language*.

[54] Mikolov T., Sutskever I., Chen K., Corrado G., Dean J., Burges C. J. C., Bottou L., Welling M., Ghahramani Z., Weinberger K. Q. (2013). Distributed representations of words and phrases and their compositionality. *Advances in Neural Information Processing Systems 26*.

[55] Bojanowski P., Grave E., Joulin A., Mikolov T. (2017). Enriching word vectors with subword information. *Transactions of the Association for Computational Linguistics*.

[56] Pennington J., Richard Socher C. D. M. GloVe: global vectors for word representation.

[57] Wei C.-P., Lee Y.-H. (2004). Event detection from online news documents for supporting environmental scanning. *Decision Support Systems*.

[58] Zhu Q., Li X., Conesa A., Pereira C. (2018). GRAM-CNN: a deep learning approach with local context for named entity recognition in biomedical text. *Bioinformatics*.

[59] Prokhorov V., Pilehvar M. T., Lio P., Collier N. Unseen word representation by aligning heterogeneous lexical semantic spaces.

[60] Makazhanov A., Rafiei D. Predicting political preference of Twitter users.

[61] Bosco C., Patti V., Bolioli A. (2013). Developing corpora for sentiment analysis: the case of irony and senti-TUT. *IEEE Intelligent Systems*.

[62] Peters M. E., Neumann M., Iyyer M. Deep contextualized word representations.

[63] Minaee S., Kalchbrenner N., Cambria E., Nikzad N., Chenaghlu M., Gao J. (2022). Deep learning--based text classification. *ACM Computing Surveys*.

[64] Zhu X., Sobihani P., Guo H. Long short-term memory over recursive structures.

[65] Zhou P., Qi Z., Zheng S., Xu J., Bao H. (2016). Text classification improved by integrating bidirectional lstm with two-dimensional max pooling. https://arxiv.org/abs/1611.06639.

[66] Wei W., Li J., Cao L., Ou Y., Chen J. (2013). Effective detection of sophisticated online banking fraud on extremely imbalanced data. *World Wide Web*.

[67] Alfuraih S. I., Sui N. T., McLeod D. (2004). Using Trusted Email to Prevent Credit Card Frauds in Multimedia Products. *World Wide Web*.

[68] Yan D., Guo S. (2019). Leveraging contextual sentences for text classification by using a neural attention model. *Computational Intelligence and Neuroscience*.

[69] Dai Z., Yang Z., Yang Y., Jaime C., Quoc V. L., Ruslan S. Transformer-XL: Attentive language models beyond a fixed-length context.

[70] Hu Y., Ding J., Dou Z., Chang H. (2022). Short-text classification detector: a bert-based mental approach. *Computational Intelligence and Neuroscience*.

[71] Devlin J., Chang M.-W., Lee K., Toutanova K. (2018). BERT: Pre-training of Deep Bidirectional Transformers for Language Understanding.

[72] Howard J., Ruder S. (2018). Universal Language Model Fine-tuning for Text Classification. https://arxiv.org/abs/1801.06146.

[73] Sun Y., Wang S., Li Y. (2019). Enhanced representation through knowledge integration. https://arxiv.org/abs/1904.09223.

[74] Varun Dogra Assvknj and M. T., Peng S.-L., Hsieh S.-Y., Gopalakrishnan S., Balaganesh (2021). Analyzing DistilBERT for sentiment classification of banking financial news. *Intelligent Computing and Innovation on Data Science*.

[75] Dogra V., Verma S., Singh A. (2021). Banking news-events representation and classification with a novel hybrid model using DistilBERT and rule-based features. *Computer Science*.

[76] Sanh V., Debut L., Chaumond J., Wolf T. (2019). DistilBERT, a distilled version of BERT: smaller, faster, cheaper and lighter. https://arxiv.org/abs/1910.01108.

[77] Ye Z., Guo Q., Gan Q., Qiu X., Zhang Z. (2019). BP-transformer: modelling long-range context via binary partitioning. https://arxiv.org/abs/1911.04070.

[78] Yamada I., Shindo H. (2019). Aip R Neural Attentive Bag-Of-Entities Model for Text Classification.

[79] Brown T. B., Mann B., Ryder N. Language models are few-shot learners.

[80] Magnini B., Strapparava C., Pezzulo G., Gliozzo A. (2002). The role of domain information in Word Sense Disambiguation. *Natural Language Engineering*.

[81] Bilenko M., Mooney R., Cohen W., Ravikumar P., Fienberg S. (2003). Adaptive name matching in information integration. *IEEE Intelligent Systems*.

[82] Huang S. J., Gao W., Zhou Z. H. (2019). Fast multi-instance multi-label learning. *IEEE Transactions on Pattern Analysis and Machine Intelligence*.

[83] Zhang M.-L., Zhou Z.-H. (2007). ML-KNN: a lazy learning approach to multi-label learning. *Pattern Recognition*.

[84] Jun Yan J., Benyu Zhang B., Ning Liu N. (2006). Effective and efficient dimensionality reduction for large-scale and streaming data preprocessing. *IEEE Transactions on Knowledge and Data Engineering*.

[85] Xu X., Liang T., Zhu J., Sun D., au T. (2019). Review of classical dimensionality reduction and sample selection methods for large-scale data processing. *Neurocomputing*.

[86] Armanfard N., Reilly J. P., Komeili M. (2016). Local feature selection for data classification. *IEEE Transactions on Pattern Analysis and Machine Intelligence*.

[87] Pölsterl S., Conjeti S., Navab N., Katouzian A. (2016). Survival analysis for high-dimensional, heterogeneous medical data: exploring feature extraction as an alternative to feature selection. *Artificial Intelligence in Medicine*.

[88] Mladenić D., Grobelnik M. (2003). Feature selection on hierarchy of web documents. *Decision Support Systems*.

[89] Haury A.-C., Gestraud P., Vert J.-P. (2011). The influence of feature selection methods on accuracy, stability and interpretability of molecular signatures. *PLoS One*.

[90] Yang J., Liu Y., Zhu X., Zhang Z., Liu Z., Zhang X. (2012). A new feature selection based on comprehensive measurement both in inter-category and intra-category for text categorization. *Information Processing & Management*.

[91] Inza I., Larrañaga P., Blanco R., Cerrolaza A. J. (2004). Filter versus wrapper gene selection approaches in DNA microarray domains. *Artificial Intelligence in Medicine*.

[92] Uysal A. K., Gunal S. (2012). A novel probabilistic feature selection method for text classification. *Knowledge-Based Systems*.

[93] Roobaert D., Karakoulas G., Chawla N. V. (2008). Information gain, correlation and support vector machines. *Feature Extraction*.

[94] Lei S. A feature selection method based on information gain and genetic algorithm.

[95] Jin X., Xu A., Bie R., Guo P. (2006). Machine learning techniques and chi-square feature selection for cancer classification using SAGE gene expression profiles. *Lecture Notes in Computer Science*.

[96] Chen Y.-T., Chen M. C. (2011). Using chi-square statistics to measure similarities for text categorization. *Expert Systems with Applications*.

[97] Gu Q., Li Z., Han J. (2010). Generalized Fisher score for feature selection. *A brief review of Fisher score. Ratio*.

[98] Weston J., Mukherjee S., Chapelle O., Pontil M., Poggio T., Vapnik V. (2000). Feature selection for SVMs. *Advances in Neural Information Processing Systems*.

[99] Youn E., Koenig L., Jeong M. K., Baek S. H. (2010). Support vector-based feature selection using Fisher’s linear discriminant and Support Vector Machine. *Expert Systems with Applications*.

[100] Wang Y., Wang X.-J. A new approach to feature selection in text classification.

[101] Genkin A., Lewis D. D., Madigan D. (2007). Large-scale bayesian logistic regression for text categorization. *Technometrics*.

[102] Peng H., Long F., Ding C. (2005). Feature selection based on mutual information criteria of max-dependency, max-relevance, and min-redundancy. *IEEE Transactions on Pattern Analysis and Machine Intelligence*.

[103] Labani M., Moradi P., Ahmadizar F., Jalili M. (2018). A novel multivariate filter method for feature selection in text classification problems. *Engineering Applications of Artificial Intelligence*.

[104] Norman K. A., Polyn S. M., Detre G. J., Haxby J. V. (2006). Beyond mind-reading: multi-voxel pattern analysis of fMRI data. *Trends in Cognitive Sciences*.

[105] Wang L., Lei Y., Zeng Y., Tong L., Yan B. (2013). Principal feature analysis: a novel voxel selection method for fMRI data. *Computational and Mathematical Methods in Medicine*.

[106] Granitto P. M., Furlanello C., Biasioli F., Gasperi F. (2006). Recursive feature elimination with random forest for PTR-MS analysis of agroindustrial products. *Chemometrics and Intelligent Laboratory Systems*.

[107] Mao K. Z. (2002). Fast orthogonal forward selection algorithm for feature subset selection. *IEEE Transactions on Neural Networks*.

[108] Chen H., Jiang W., Li C., Li R. (2013). A heuristic feature selection approach for text categorization by using chaos optimization and genetic algorithm. *Mathematical Problems in Engineering*.

[109] Leardi R., Boggia R., Terrile M. (1992). Genetic algorithms as a strategy for feature selection. *Journal of Chemometrics*.

[110] Guyon I., Bitter H.-M., Ahmed Z., Brown M., Heller J. Multivariate non-linear feature selection with kernel multiplicative updates and gram-schmidt relief.

[111] Urbanowicz R. J., Meeker M., Cava W. L, Olson R. S., Moore J. H. (2018). Relief-based feature selection: introduction and review. *Journal of Biomedical Informatics*.

[112] Urbanowicz R. J., Olson R. S., Schmitt P., Meeker M., Moore J. H. (2018). Benchmarking relief-based feature selection methods for bioinformatics data mining. *Journal of Biomedical Informatics*.

[113] Mimouni N., Yeung T. Y. Comparing Performance of Text Pre-processing Methods for Predicting a Binary Position by LASSO Experiment with Textual Data of European Union Public Consultation.

[114] Marafino B. J., Boscardin W. J., Dudley R. A (2015). Efficient and sparse feature selection for biomedical text classification via the elastic net: application to ICU risk stratification from nursing notes. *Journal of Biomedical Informatics*.

[115] Taylor P., Hoerl A. E., Kennard R. W. (2012). *Technometrics Ridge Regression: Biased Estimation for Nonorthogonal Problems Ridge Regression : Biased Estimation Nonorthogonal Problems*.

[116] Zou H., Hastie T. (2005). Regularization and variable selection via the elastic net. *Journal of the Royal Statistical Society: Series B*.

[117] Sebastiani F. (2002). Machine learning in automated text categorization. *ACM Computing Surveys*.

[118] Gomez J. C., Moens M. F. (2012). PCA document reconstruction for email classification. *Computational Statistics & Data Analysis*.

[119] Blei D. M., Ng A. Y., Jordan M. I. (2003). *Latent Dirichlet Allocation*.

[120] Ordun C., Purushotham S., Raff E. (2020). Exploratory Analysis of Covid-19 Tweets Using Topic Modeling, UMAP, and DiGraphs. https://arxiv.org/abs/2005.03082.

[121] Mcinnes L., Healy J., Melville J. (2020). UMAP: Uniform Manifold Approximation and Projection for Dimension Reduction. https://arxiv.org/abs/1802.03426.

[122] Rehman H. U., Shafiq M., Baig S., Manzoor U. (2021). Analyzing the epidemiological outbreak of COVID-19. *A Vis Explor data Anal approach J Med Virol*.

[123] Cheng H., Yu R. (2021). *Text Classification Model Enhanced by Unlabeled Data for LaTeX Formula*.

[124] Chen J., Huang H., Tian S., Qu Y. (2009). Feature selection for text classification with Naïve Bayes. *Expert Systems with Applications*.

[125] Trstenjak B., Mikac S., Donko D. (2014). KNN with TF-IDF based framework for text categorization. *Procedia Engineering*.

[126] Lee C., Lee G. G. (2006). Information gain and divergence-based feature selection for machine learning-based text categorization. *Information Processing & Management*.

[127] Khreisat L. (2009). A machine learning approach for Arabic text classification using N-gram frequency statistics. *Journal of Informetrics*.

[128] Liu J., Jin T., Pan K., Yang Y., Wu Y., Wang X. An improved KNN text classification algorithm based on Simhash.

[129] Apté C., Weiss S. (1997). Data Mining with Decision Trees and Decision Rules. *Future Generation Computer Systems*.

[130] Phu V. N., Tran V. T. N., Chau V. T. N., Duy N. D., au K. L. D. (2017). A decision tree using ID3 algorithm for English semantic analysis. *International Journal of Speech Technology*.

[131] Mahender C. N. (2012). *TEXT CLASSIFICATION AND CLASSIFIERS*.

[132] Yang Y. (1997). An evaluation of statistical approaches to text categorization. *Inf Retr Boston*.

[133] Roe B. P., Yang H. J., Zhu J., Liu Y., Stancu I., McGregor G. (2005). Boosted decision trees as an alternative to artificial neural networks for particle identification. *Nuclear Instruments and Methods in Physics Research Section A: Accelerators, Spectrometers, Detectors and Associated Equipment*.

[134] Ye J., Chow J. H., Chen J., Zheng Z. Stochastic gradient boosted distributed decision trees.

[135] Wiener E., Pedersen J., Weigend A. A neural network approach to topic spotting.

[136] Johnson R., Zhang T. (2014). Effective Use of Word Order for Text Categorization with Convolutional Neural Networks. https://arxiv.org/abs/1412.1058.

[137] Zhang Y., Zhang Z., Miao D., Wang J. (2019). Three-way enhanced convolutional neural networks for sentence-level sentiment classification. *Information Sciences*.

[138] Kalchbrenner N., Grefenstette E., Blunsom P. A convolutional neural network for modelling sentences.

[139] Elman J. L. (1990). Finding structure in time. *Cognitive Science*.

[140] Makarenkov V., Guy I., Hazon N., Meisels T., Shapira B., Rokach L. (2019). Implicit dimension identification in user-generated text with LSTM networks. *Information Processing & Management*.

[141] Sarmento L., Carvalho P., Silva M. J., de Oliveira E. (2009). Automatic creation of a reference corpus for political opinion mining in user-generated content. *Attitude*.

[142] Ruchansky N., Seo S., Liu Y. CSI: A Hybrid Deep Model for Fake News Detection.

[143] Dong S., Liu C. (2021). Sentiment classification for financial texts based on deep learning. *Computational Intelligence and Neuroscience*.

[144] Zhu Y., Zheng W., Tang H. (2020). Interactive dual attention network for text sentiment classification. *Computational Intelligence and Neuroscience*.

[145] Chung J. (2014). Gated Recurrent Neural Networks on Sequence Modeling. https://arxiv.org/abs/1412.3555.

[146] Tang D., Qin B., Liu T. Document modeling with gated recurrent neural network for sentiment classification.

[147] Zulqarnain M., Ghazali R., Ghouse M. G., Mushtaq M. F. (2019). Efficient processing of GRU based on word embedding for text classification. *JOIV: International Journal on Informatics Visualization*.

[148] Kuchaiev O., Ginsburg B. Factorization tricks for LSTM networks.

[149] Shazeer N., Mirhoseini A., Maziarz K. (2017). Outrageously large neural networks: the sparsely-gated mixture-of-experts layer. https://arxiv.org/abs/1701.06538.

[150] Jain B. C. W. (2019). Attention is not Explanation. https://arxiv.org/abs/1902.10186.

[151] Serrano S., Smith N. A. Is attention interpretable?.

[152] Vashishth S., Upadhyay S., Tomar G. S., Faruqui M. (2019). Attention Interpretability Across NLP Tasks. https://arxiv.org/abs/1909.11218.

[153] Munkhdalai T., Yu H. Neural semantic encoders.

[154] Commissariat T. (2020). Ask me anything. *Physics World*.

[155] Vaswani A., Shazeer N., Parmar N. Attention is all you need.

[156] Sun C., Qiu X., Xu Y., Huang X. (2019). How to fine-tune BERT for text classification?. *Lect Notes Comput Sci (Including Subser Lect Notes Artif Intell Lect Notes Bioinformatics)*.

[157] Lan Z., Chen M., Goodman S., Gimpel K., Sharma P., Soricut R. (2019). ALBERT: A Lite BERT for Self-supervised Learning of Language Representations. https://arxiv.org/abs/1909.11942.

[158] Joshi M., Chen D., Liu Y., Weld D. S., Zettlemoyer L., Levy O. (2019). SpanBERT: Improving Pre-training by Representing and Predicting Spans. https://arxiv.org/abs/1907.10529.

[159] Holzhey C., Larsen F., Wilczek F. (1994). Geometric and renormalized entropy in conformal field theory. *Nuclear Physics B*.

[160] Nigam K., Lafferty J., Mccallum A. (1999). Using maximum entropy for text classification. *Computet Science*.

[161] Atkinson-Abutridy J., Mellish C., Aitken S. (2004). Combining information extraction with genetic algorithms for text mining. *IEEE Intelligent Systems*.

[162] Bhardwaj A., Narayan Y., Vanraj, Pawan, Dutta M. (2015). Sentiment analysis for Indian stock market prediction using sensex and nifty. *Procedia Computer Science*.

[163] Kim D., Seo D., Cho S., Kang P. (2019). Multi-co-training for document classification using various document representations: TF-IDF, LDA, and Doc2Vec. *Information Sciences*.

[164] Steinberg M. H. (2008). Clinical trials in sickle cell disease: adopting the combination chemotherapy paradigm. *American Journal of Hematology*.

[165] Xu Z., Sun J. (2018). Model-driven deep-learning. *National Science Review*.

[166] Chen Y., Xiao B., Lin Z., Dai C., Li Z., Yan L. Multi-label text classification with deep neural networks.

[167] Pereira R. B., Plastino A., Zadrozny B., Merschmann L. H. C. (2018). Categorizing feature selection methods for multi-label classification. *Artificial Intelligence Review*.

[168] Stein R. A., Jaques P. A., Valiati J. F. (2019). An analysis of hierarchical text classification using word embeddings. *Information Sciences*.

[169] Sinoara R. A., Sundermann C. V., Marcacini R. M., Domingues M. A., Rezende S. R. Named Entities as Privileged Information for Hierarchical Text Clustering.

